# Knockdown of endothelial *Serpine1* improves stroke recovery by attenuating peri-infarct blood flow and blood-brain barrier disruption

**DOI:** 10.1038/s41419-026-09062-9

**Published:** 2026-06-27

**Authors:** Kamal Narayana, Isabel C. Lambert, Sam Burford, Emilie Gosselin, Jakob Körbelin, Craig E. Brown

**Affiliations:** 1 Division of Medical Sciences, University of Victoria, Victoria, BC Canada; 2 Department of Oncology, Hematology and Bone Marrow Transplantation, University Medical Center Hamburg-Eppendorf, Hamburg, Germany; 3 Department of Psychiatry, University of British Columbia, Vancouver, BC Canada

**Keywords:** Stroke, Blood-brain barrier, Blood flow

## Abstract

Focal stroke leads to complex changes in the cerebral microcirculation in surviving brain tissues that strongly influence functional recovery. The gene *Serpine1* and its protein product Plasminogen Activator-Inhibitor-1 (PAI-1), are highly upregulated in endothelial cells after stroke, are known to inhibit clot breakdown and are an established biomarker of cardiovascular disease in humans. Therefore, we hypothesized that inhibiting this pathway specifically within brain endothelial cells could be beneficial for stroke recovery by promoting fibrinolysis and cerebral blood flow (CBF). Using longitudinal in vivo imaging, we first show in wild-type mice that focal ischaemic stroke leads to a transient reduction in peri-infarct CBF at 6 h (~41%), which is then followed by hyperperfusion at 3 days (~29%). Contrary to expectation, viral knockdown of *Serpine1* in brain endothelial cells led to a persistent reduction in peri-infarct capillary width (~20–37%) and red blood cell velocity (~36–50%) over this time. Consistent with this effect on CBF, topical application of PAI-1 increased capillary diameter and flow. Of note, lowered peri-infarct CBF in *Serpine1* knockdown mice appeared to play a protective role in stroke recovery since it attenuated deleterious blood-brain barrier disruption and pro-inflammatory gene expression. In agreement with this, *Serpine1* knockdown mice displayed enhanced recovery of sensory evoked cortical responses, as well as improved cognitive and sensorimotor function relative to wild-type mice. These findings suggest that endothelial *Serpine1/PAI-1* signalling can influence vessel tone and highlight its therapeutic potential in promoting stroke recovery. Further, our data challenge the assumption that increased CBF after the hyper-acute phase of stroke is better for recovery and suggest that carefully tuning flow, rather than maximizing it, may be an optimal strategy.

## Introduction

**Fig. 1:**
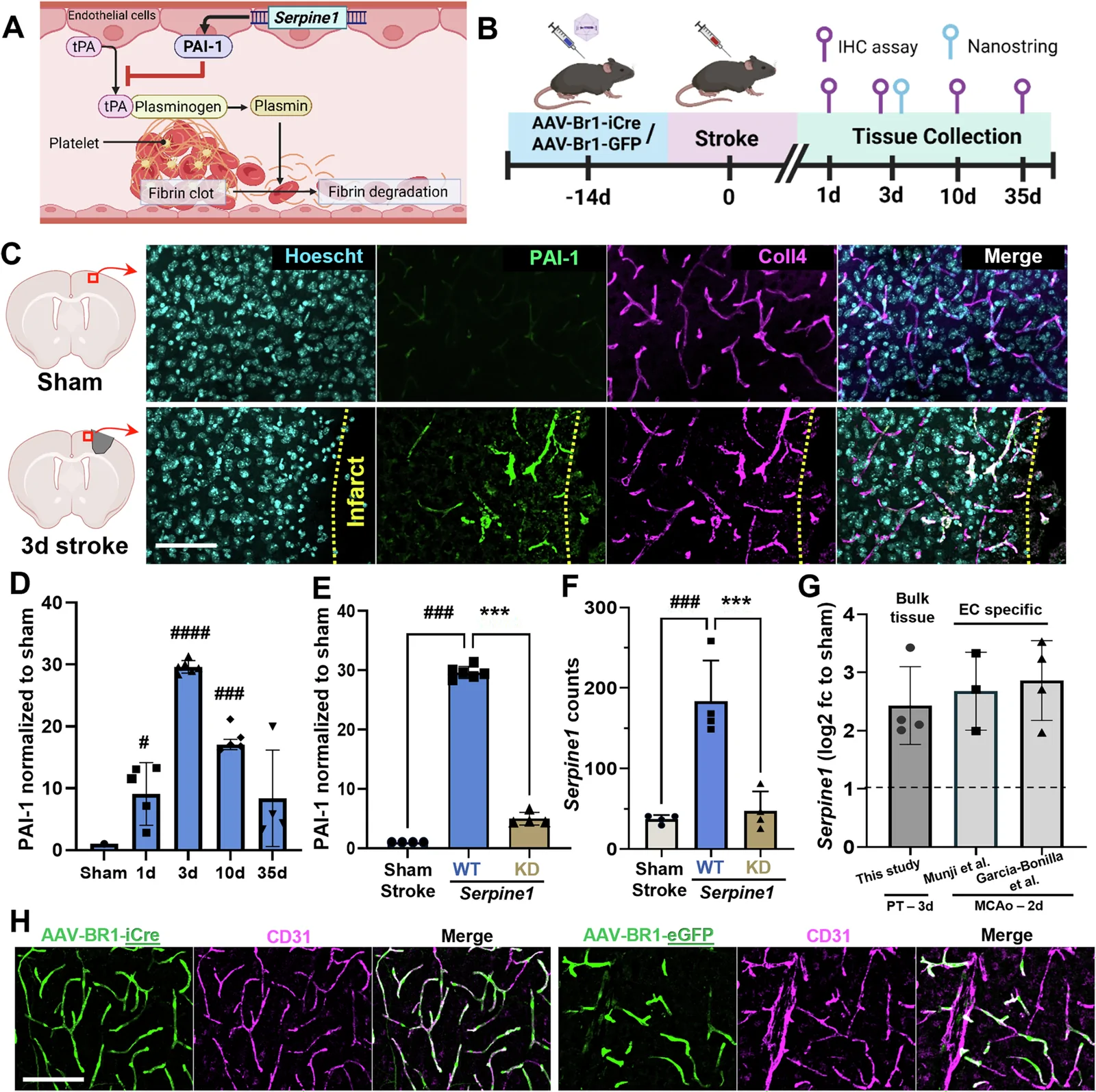
*Serpine1*/PAI-1 is upregulated in peri-infarct cortex. **A** Summary diagram showing PAI-I regulation of thrombolysis. **B** Timeline of experiments examining *Serpine1*/PAI-1 changes after stroke. **C** Confocal immunofluorescent images of PAI-1 (green), Hoescht (cyan), and Collagen4 (purple) in the peri-infarct region (PI; region left of yellow border) in sham stroke control and 3 d post-stroke. Scale bar = 100 µm. **D** PAI-1 immunostaining area normalized to sham control in peri-infarct regions in sham (*n* = 4), 1 d = (*n* = 4), 3 d (*n* = 5), 10 d (*n* = 4), and 35 d (*n* = 4). One-sample t-tests compared post-stroke day to sham. **E** PAI-1 immunostaining area normalized to sham control in peri-infarct cortex of sham (no stroke), *Serpine1* WT and KD at 3 d post-stroke. One-sample t-tests were used to compare stroke to sham controls, while an unpaired two-tailed t-test was used to compare *Serpine1* WT and KD. **F** *Serpine1* gene counts in peri-infarct cortex of sham (no stroke), *Serpine1* WT and KD at 3 d post-stroke (*n* = 4/group). Data analysed with one-way ANOVA & Tukey’s multiple comparisons. **G** Comparison of *Serpine1* gene expression (based on Log_2_ fold change vs sham controls) at 3 d post-stroke (this study) and two separate studies examining endothelial cell (EC) expression at 2 d post-stroke. Models of stroke: PT – photothrombotic stroke (this study), MCAo – middle cerebral artery occlusion (Munji et al. (*n* = 3), and Garcia-Bonilla et al. (*n* = 4)). **H** Confocal images in somatosensory cortex showing Cre-dependent TdTomato reporter expression in endothelial cells (co-labelled with CD31) after injection of AAV-BR1-iCre (left panel) or endothelial eGFP expression after injection with AAV-BR1-eGFP (right panel). Scale bar = 100 µm. Comparisons between *Serpine1* KD vs WT: *** *p* < 0.001. Comparisons between stroke vs. sham stroke mice: #### *p* < 0.0001, ### *p* < 0.001, # *p* < 0.05. Data expressed as the mean ± SD.

Stroke is a cerebrovascular disease that results in irreversible cell death in the ischaemic core as well as chronic dysfunction in surviving brain regions [1]. As a result, patients who survive a stroke will likely face permanent disabilities that affect their quality of life. Regulating blood flow in vulnerable brain tissues within the first few h to weeks after stroke is considered a critical factor in functional recovery [2–4]. Signalling pathways involved in fibrinolysis, such as the *Serpine1* gene and its effector protein Plasminogen Activator-Inhibitor-1 (PAI-1), are involved in regulating blood flow after stroke. PAI-1 is the primary physiological inhibitor of tissue plasminogen activator (tPA) and urokinase-PA (uPA) in the fibrinolysis cascade. PAI-1 prevents tPA from converting plasminogen to plasmin, effectively preventing the breakdown of fibrinous clots (Fig. 1A). PAI-1 levels rapidly rise in blood and brain after stroke [5], primarily produced from endothelial cells in mice, and to a lesser extent in platelets and microglia [6–10]. High levels of plasma PAI-1 are a risk factor for ischaemic stroke and reinfarction (i.e., secondary stroke) in human patients [11–17]. In addition to regulating fibrinolysis, a much lesser-known function of endothelial *Serpine1*/PAI-1 is to influence blood vessel tone, at least in heart smooth muscle [18, 19]. Thus, *Serpine1*/PAI-1 signalling is positioned to potentially regulate both coagulation and vessel tone, raising the intriguing possibility that blocking *Serpine1*/PAI-1 signalling could be a promising strategy for altering blood flow in a manner that optimizes outcomes after stroke. Furthermore, given its modulatory role in the anti-coagulant pathway, some of the deleterious side effects of thrombolytics may be avoided by inhibiting *Serpine1*/PAI-1 signalling.

There are pre-clinical animal studies that have systemically modulated PAI-1 activity after stroke, but with mixed findings. For example, the protective effects of *Serpine1*/PAI-1 have been shown, given that congenital *Serpine1* knockout mice have significantly larger infarcts after focal ischaemic stroke, while PAI-1 over-expression reduces the extent of infarction [13, 20–24]. Contrasting with this, other studies have reported a deleterious role since PAI-I can potentiate neurovascular damage after brain trauma [25]. Further, systemic inhibition of PAI-1 with knockout mice or intravenous injection of blocking antibodies led to improved blood flow in large surface vessels, enhanced behavioural measures of recovery and lowered infarct volumes after ischaemic stroke [13, 26, 27]. However, these studies were limited by the fact that they did not address blood flow or vascular leakage at the microvascular level, and used non-cell or tissue-specific manipulations of Serpine1/PAI-I. Thus, it remains uncertain whether a cell-specific manipulation of *Serpine1*/PAI-1 alters microvascular blood flow, influences blood-brain barrier (BBB) permeability, or affects functional recovery. In the present study, we focused on inhibiting *Serpine1*/PAI-1 signalling specifically within brain endothelial cells. To do this, we longitudinally imaged microvascular networks in vivo before and after photothrombotic stroke in mice with viral-mediated knockdown of endothelial *Serpine1*. Contrary to expectations, we found a long-lasting reduction in peri-infarct blood flow in *Serpine1* knockdown mice. Reduced blood flow attenuated BBB dysfunction and enhanced recovery of sensory-evoked cortical activity, sensori-motor and cognitive function.

## Materials and methods

For a detailed description of methods pertaining to surgery, IOS imaging, histology, behavioral testing, Nanostring and cytokine assays, see Supplemental data file.

### Experimental animals and exclusion criteria

Adult (2–4 month old) female and male *Serpine1*^fl/fl^ mice on C57BLJ background (Jackson #032522) were used in all experiments. Although male and female mice were included in each experiment, we did not have sufficient sampling of each sex and statistical power to draw conclusions about possible sex differences. *Serpine1* floxed mice were bred to homozygosity (*Serpine1*^fl/fl^), and offspring were genotyped using the following primers: Wild-type; 5’–CAC-CAT-GAA-AGG-GTA-CCA-CTG-TCA-G–3’, Floxed; 5’ GCG-CCG-GAA-CCG-AAG-T 3’. Mice were housed under a 12-h light/dark cycle and given *ad libitum* access to water and laboratory diet (Picolab Rodent diet 20, #50553). Ventilated housing racks were controlled for temperature (21–23 °C) and humidity (40–50%). All experiments were conducted according to the guidelines set by the Canadian Council of Animal Care and approved by the University of Victoria Animal Care Committee (Animal use protocol #: AE-24-015-05). Sample sizes for each experiment were similar to previous studies from our lab, based on expected variances associated with each dependent measure. Animals were excluded from the study (see Supplementary Table 1) if they met any of the following criteria: [1] had their cranial window detach during the experimental period, [2] had an infarct that destroyed tissue imaged before stroke or disrupted the optical clarity of the cranial window, [3] reached a humane endpoint criteria in accordance with our institutional ethical guidelines.

### AAV-based knockdown of *Serpine1* in brain endothelial cells

We used the AAV-BR1 serotype for expressing Cre recombinase in endothelial cells of *Serpine1*^fl/fl^ mice [28]. Mice were randomly assigned (based on a coin flip) to either control or knockdown groups. Experimenters were blind to the control or knockdown treatment groups. However, experimenters performing brain imaging could not be blind to the stroke condition since it was obvious and essential for determining imaging locations. *Serpine1*^fl/fl^ mice were anaesthetized with ~1.5% isofluorane in medical air and intravenously injected with 20 µL of AAV-BR1-iCre (5.0 × 10^12^ GC/mL). Wild-type controls consisted of *Serpine1*^fl/fl^ mice injected with equivalent doses of AAV-BR1-eGFP.

### Photothrombotic stroke

We performed a targeted ischaemic stroke to the right forelimb (FL) somatosensory cortex (mapped from IOS imaging) using the photothrombotic method [29, 30]. All mice were anaesthetized with ~1.3–1.5% isofluorane in medical air and fitted to a custom imaging stage under an upright Olympus BX51WI microscope. Animal body temperature was maintained at 37 °C throughout the procedure. For mice that did not already have a cranial window installed (this only applies to experiments shown in Supplementary Fig. 3, as well as 50% of mice used for behavioural analysis), they first received an injection of lidocaine under the scalp before the scalp was cut and retracted. The skull was carefully thinned over the right FL somatosensory cortex with a high-speed dental drill. Mice with cranial windows already installed did not receive any perioperative pain management. All mice were administered 1% Rose Bengal solution (100 mg/kg in HEPES-buffered ACSF) via intraperitoneal (i.p.) injection. For targeting stroke to the right FL cortex, it was illuminated with a green LED (wavelength centred at 532 nm) through a 10x objective lens, covering a 1 mm diameter region for 15–20 min. For the experiment where we directly targeted a branch of the MCA (see Supplementary Fig. 3), we thinned a small portion of the skull directly over the MCA, then masked the surrounding skull surface with black marker and directed a green laser (532 nm, 12 mW) to the MCA branch until flow stopped. The sham stroke group received either an injection of 1% Rose Bengal but no illumination, or vice versa.

### In vivo two-photon imaging of capillary blood flow

Mice were lightly anaesthetized with 1% isofluorane in medical air and fitted to a custom imaging stage. Mice were intravenously injected with 2% FITC dextran for labelling blood plasma (Sigma, 46945, molecular weight 70 kDa, in 0.9% saline). High-resolution images of cerebral vasculature were acquired using an Olympus FV1000MPE laser scanning microscope fed by a mode-locked Ti: Sapphire laser source with an average delivered laser power of 15 to 50 mW at 800 nm wavelength. Emitted light was split by a dichroic filter (552 nm) before it passed through either a 495–540 nm or 558–706 nm bandpass filter. Image stacks were acquired to a depth of ~250 µm below the pial surface with a 20x Olympus XLUPlanFl water-immersion objective (NA = 0.95) using Olympus Fluoview FV10-ASW software. Stacks were collected at a zoom of 1.3, z-step size of 1.25μm, covering an area of 489.5×489.5 µm (0.48μm/pixel). Cortical areas close to (range from 113 to 352 µm) and further away (range from 491 to 1643 µm) from the infarct border were imaged and relocated at respective days using local vessel landmarks and reference coordinates.

To measure RBC velocity, we collected line scans through the middle of several different capillary segments and repeated this three times with 30 s between each scan to obtain the average RBC velocity for a capillary. Since moving RBCs are unlabelled, the resulting line scan has angled streaks where the velocity is proportional to the slope of the RBC streaks. To automate our analysis of line scans, we employed particle image velocimetry (LS-PIV) MATLAB code [31]. LS-PIV calculates the RBC velocity by determining the RBC displacement between pairs of the same line-scan using spatial cross-correlation analysis. This enabled us to measure a wide range of RBC velocities at varying conditions. We then convert the detected pixel shift (also the Δ distance in manual processing) to a velocity expressed in mm/s for each individual line scan. Vessel width was measured using the VasoMetrics macro on ImageJ/Fiji [32]. In general, we collected fluorescence intensity profiles at 5 µm intervals through a capillary across each branch order, starting from the 1st branch to the 6th. Diameter was then estimated based on the average distance between the two half-maximum intensity points on the Gaussian-like intensity profile.

### Quantification of BBB permeability

For assessing BBB permeability in vivo, mice were prepared for imaging as described above and intravenously injected with 75 µL of 2.5% lower molecular weight FITC dextran (4 kDa, Sigma, 46944, in 0.9% saline). Two-photon imaging (wavelength set to 800 nm) started within 5 min after FITC-dextran injection. Image stacks consisted of 10 images spanning 424×424 µm (Kalman average = 2), collected at 10 µm z-steps from a depth of 0–100 µm from the cortical surface. These stacks were taken under identical laser power/imaging conditions, every 2 min for up to 30 min. FITC-dextran extravasation was measured based on fluorescence intensity on a 12-bit scale, by placing an ROI (15×15 µm) in the extravascular space (minimum ~5 µm from the nearest vessel). Changes in extravascular fluorescence intensity at each time point were normalized to the first time point (0 min).

For post-mortem assessment of BBB permeability 3 days after stroke (see Supplementary Fig. 3), mice were injected (i.v.) with 3% Evans Blue dye dissolved in saline. Dye was allowed to circulate for 45 min, then mice were deeply anesthetized and perfused with 0.1 M PBS. Brains were extracted and post-fixed overnight. Immediately after cutting on a vibratome (100 µm thick), sections were mounted onto slides and imaged using a confocal microscope. Images of Evans blue dye extravasation were collected with a 10X objective (NA = 0.40) using a 635 nm laser and Cy5 emission filters. Image stacks were collected with a Kalman filter (average = 2 frames) in 4 µm Z-steps with pixel sampling in x-y set at 1.41 µm/pixel. Extravascular Evans blue fluorescence in peri-infarct cortex was then normalized to the contralateral hemisphere.

### Statistics

Statistical analysis of the data was conducted using R/R-studio (Version 4.4.1) and GraphPad Prism (version 10.4.2). Normality of continuous variables in all figures was assessed using the Shapiro-Wilk and Kolmogorov-Smirnov tests; all distributions had *p* > 0.05 and were thus considered normally distributed, justifying the use of parametric tests. One-way analysis of variance (ANOVA) was used to compare baseline measurements of vascular parameters (blood flow and vessel width) across experimental groups. Planned two-sample unpaired t-tests were used for comparing groups of 3 or less. One-way ANOVA followed by Sidak comparisons was used for analysing PAI-1 staining over time. Two-way ANOVA was used to analyse effects of stroke, genotype (*Serpine1* knockdown), or interaction effects on RBC velocity, flux, vessel width, BBB leakage, IOS responses, cytokine expression levels and behaviour. Significant main effects or interactions were followed up by *post-hoc* multiple comparisons (Tukey, or Mann-Whitney on the data set). For Nanostring experiments, a moderated t-test with multiple test corrections using limma was used to determine significance in gene expression. Transcriptomic plots, including volcano plots, heatmaps, horizontal bar graphs, and line plots, were made using R/R-studio. RBC velocities from line scans were acquired using MathLab R2023a.

## Results

### Stroke upregulates *Serpine1* and PAI-1 expression in peri-infarct cortex

We first characterized the expression of *Serpine1* mRNA and PAI-1 protein after photothrombotic stroke [29] in forelimb somatosensory cortex in male and female wild-type (WT) mice (Fig. 1B). In sham stroke mice, very little cortical PAI-1 protein (Fig. 1C–E) or *Serpine1* mRNA (Fig. 1F) was expressed. However, stroke led to a significant increase in *Serpine1* and PAI-1 expression in peri-infarct cortex that peaked 3 days after stroke (Fig. 1C–F). Since our PAI-1 immunostaining showed vascular expression (Fig. 1C), but our gene expression assay was based on bulk tissue, we also examined *Serpine1* gene expression in published single-cell RNAseq datasets collected after ischaemic stroke [6, 7]. These databases confirm that *Serpine1* is significantly upregulated in peri-infarct endothelial cells 2 days after middle cerebral artery (MCA) occlusion (Fig. 1G).

Since stroke increases *Serpine1*/PAI-1 expression in peri-infarct endothelial cells, we knocked down *Serpine1* in brain endothelial cells. To do this, *Serpine1* floxed mice were injected (i.v.) with AAV-BR1-iCre (“*Serpine1* KD”) or control virus (AAV-BR1-eGFP, “*Serpine1* WT”) two weeks before the induction of stroke (Fig. 1B). Previous research has shown that AAV-BR1 infects endothelial cells in the brain but not other cell types like microglia [28] Agreeing with previous work [33], injection of AAV-BR1-iCre into TdTomato reporter mice (Ai9 strain) leads to cre-recombination in approximately 93.7 ± 5.4% of cortical endothelial cells, while AAV-BR1-eGFP induced detectable eGFP expression in approximately 63.4 ± 17.2% of endothelial cells (see Fig. 1H). Importantly, PAI-1 and *Serpine1* expression were significantly decreased in peri-infarct tissues from *Serpine1* KD mice (Fig. 1E, F). Furthermore, we functionally validated the knockdown of *Serpine1* in cerebral capillaries by inducing clots with targeted 2-photon irradiation (Supplementary Fig. 1). In wild-type mice, all laser-targeted capillaries remained clotted at 6 h whereas only 41.7% remained clotted in *Serpine1* KD mice (χ^2^ [1, 34] = 7.66, *p* = 0.006). Collectively, these experiments validate AAV-mediated *Serpine1* knockdown.

### Endothelial *Serpine1* knockdown reduces capillary blood flow in peri-infarct cortex

**Fig. 2:**
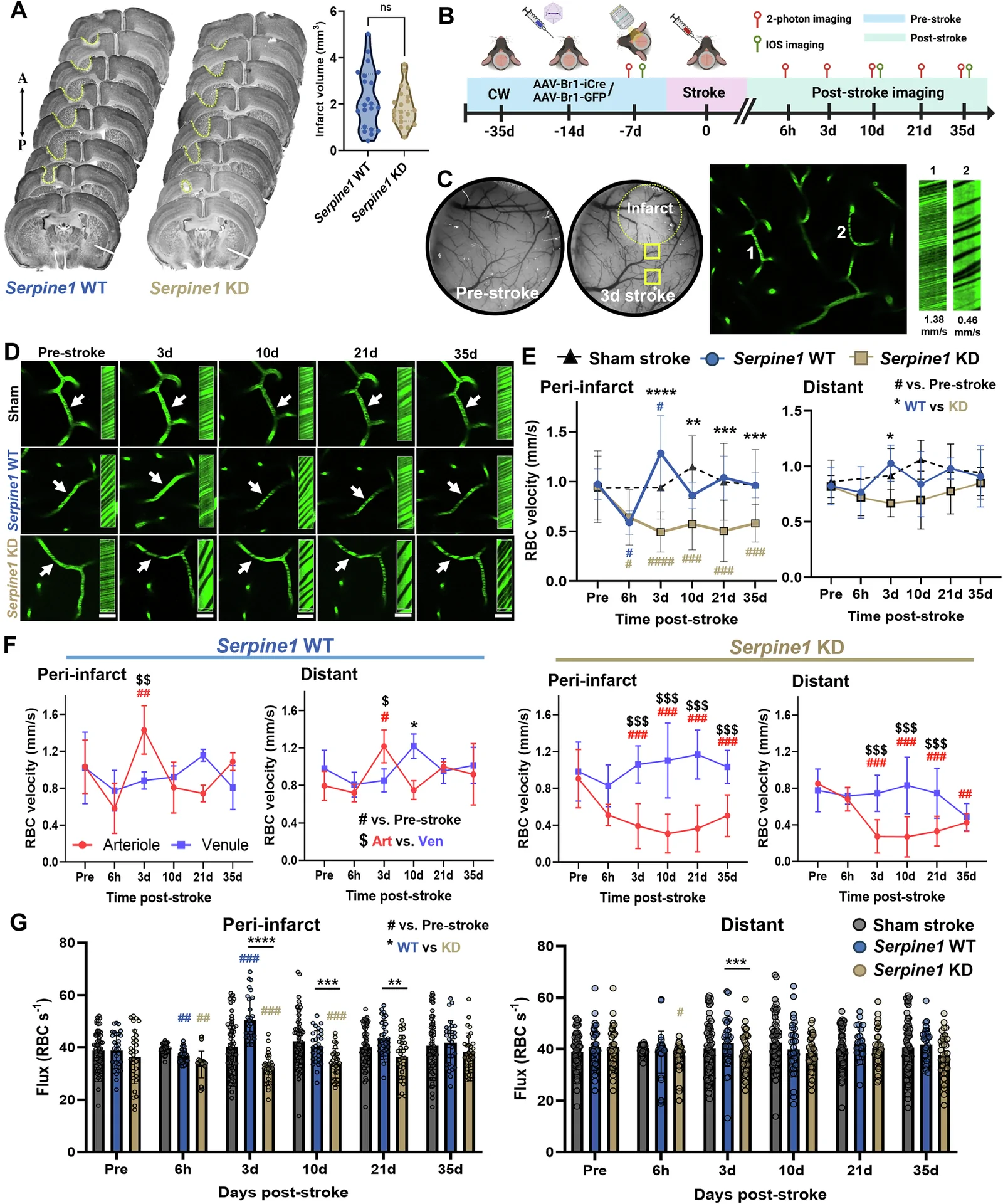
*Serpine1* KD leads to reduced blood flow velocity and flux in peri-infarct regions. **A** Infarcts (denoted by yellow border) were measured in *Serpine1* WT (blue) and *Serpine1* KD (golden) mice 3 days after stroke (*n* = 7/group). Data analysed with two-tailed unpaired *t*-test; ns, *p* = 0.51. **B** Timeline of longitudinal in vivo imaging experiments. **C** Brightfield image showing the brain surface before and 3 days after stroke. Boxed regions show the location of imaging. A maximum intensity z-projection image shows 2 capillaries and line scans; one fast flowing (1.38 mm/s), and the other slow (0.46 mm/s). **D** In vivo maximum z-projection images illustrating blood flow changes across the 35-day imaging period. Scale bar = 50 µm. **E** RBC velocity measurements in capillaries from sham control or peri-infarct and distant regions across a 35-day stroke recovery period. *N* = 6 sham stroke mice, *N* = 10 and 17 stroke-affected *Serpine1* WT and KD mice, respectively. **F** RBC velocity measurements in stroke-affected *Serpine1* WT and KD capillaries binned by proximity to nearest penetrating arteriole (red lines) or ascending venule (purple lines). **G** RBC flux (RBCs/s) in capillaries from sham stroke controls or stroke-affected *Serpine1* WT (blue) and KD mice (golden) over time. 81 capillaries were measured in each group. Data in **E**–**G** were analysed with two-way ANOVA followed by Tukey’s multiple comparisons test. Comparisons between *Serpine1* KD vs WT: **** *p* < 0.0001, *** *p* < 0.001, ** *p* < 0.01, * *p* < 0.05. Comparisons between post-stroke time-point to their respective pre-stroke (0 day) value: #### *p* < 0.0001, ### *p* < 0.001, ## *p* < 0.01, # *p* < 0.05. Comparisons between capillaries arising from the Arteriole vs Venule: $$$ *p* < 0.001, $$ *p* < 0.01, $ *p* < 0.05. Data expressed as the mean ± SD.

We next investigated the role endothelial *Serpine1*/PAI-1 signalling plays in stroke damage and recovery. First, we analysed infarct volume (at 3 days) and found no differences between *Serpine1* WT and KD mice (Fig. 2A). Given this, we next focused on microvascular blood flow dynamics that evolve over days and weeks after stroke [34]. To do this, we implanted cranial windows in homozygous *Serpine1* floxed mice and randomly assigned them to receive AAV-BR1-iCre or GFP (Fig. 2B). Under light isofluorane anaesthesia, we imaged blood flow dynamics before and after the induction of stroke (Fig. 2B–D) in both peri-infarct cortex and more distant regions (184 ± 21 µm vs. 849 ± 58 µm from infarct border, respectively). Sham stroke controls consisted of both *Serpine1* WT and KD mice (*n* = 6 total with 3 mice from each genotype) since *Serpine1*/PAI-1 expression is extremely low in the absence of stroke. In sham stroke mice, RBC velocities did not change significantly over the 35-day imaging period regardless of whether mice were injected with AAV-BR1-eGFP or iCre (dashed line in Fig. 2E and Supplementary Fig. 2). Six h after stroke induction, RBC velocity was similarly reduced between *Serpine1* WT and KD mice (38 vs 32% reduction, respectively, Fig. 2E). However, RBC velocities showed strikingly different patterns between genotypes from 3 days onwards (Fig. 2E), especially in peri-infarct cortex (2-way ANOVA, Main effect of Genotype: F_(1, 147)_ = 63.5, *p* < 0.0001; Main effect of Time: *F*_(5, 147)_ = 5.95 *p* < 0.0001; Genotype x Time Interaction: *F*_(5, 147)_ = 10.24, *p* < 0.0001), and to a lesser degree in distant regions (2-way ANOVA, Main effect of Genotype: *F*_(1, 148)_ = 31.9, *p* < 0.001; Main effect of Time: *F*_(3.1, 158.1)_ = 4.81 *p* < 0.01; Genotype x Time Interaction: *F*_(5,250)_ = 5.3, *p* < 0.001). In WT mice, RBC velocities increased in peri-infarct cortex by 3 days post-stroke (Fig. 2E), then returned to baseline levels. By contrast, peri-infarct RBC velocities in *Serpine1* KD mice remained significantly reduced from 3 days onwards (reduction ranged from 38–47%, see gold hashtags in Fig. 2E). Given well-known differences in capillary contractility across the vascular tree [35, 36], we grouped velocity measurements based on whether capillaries branched off the nearest penetrating arteriole (PA) or ascending venule (AV). Following stroke, the biggest changes in RBC velocity originated from capillaries proximal to the PA in both peri-infarct and distant regions (Fig. 2F), especially in *Serpine1* KD mice (right panel in Fig. 2F). And finally, we examined RBC flux (# RBC/s) and found significantly lower levels in peri-infarct regions in *Serpine1* KD mice from 3 to 21 days relative to *Serpine1* WT mice (Fig. 2G). These results indicate that endothelial *Serpine1 KD* reduces RBC velocity and flux in peri-infarct cortex.

### Endothelial *Serpine1* knockdown induces prolonged capillary constriction after stroke

**Fig. 3:**
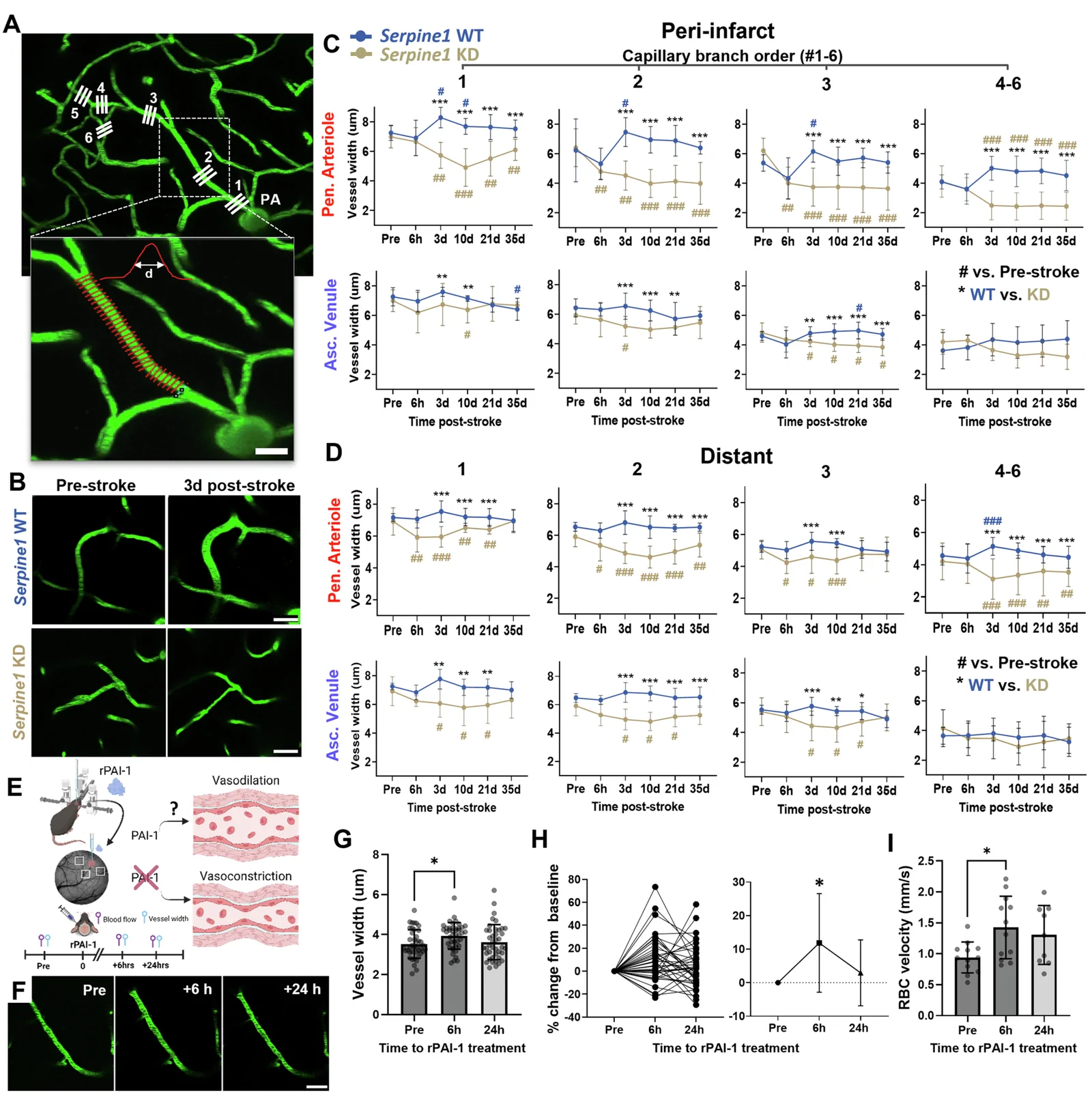
Endothelial *Serpine1* KD leads to prolonged constriction in peri-infarct capillaries. **A** In vivo maximum intensity z-projection images showing a penetrating arteriole (PA) and downstream branches from 1st to 6th order. Boxed region shows example of capillary diameter (d) measurements using Vasometrics tool in ImageJ/Fiji where diameter is estimated based on half-maximal intensity from linear plots of intravascular fluorescence. Scale bar = 50 µm. **B** Representative maximum intensity z-projection images showing that peri-infarct capillaries dilate 3 days after stroke in *Serpine1* WT mice but constrict in *Serpine1* KD mice. Scale bar = 30 µm. **C** Graphs show capillary widths in peri-infarct cortex of *Serpine1* WT (blue) and KD (golden) mice based on their branch order and whether they arise from the PA or AV. **D** Capillary widths in the distant cortex of *Serpine1* WT (blue) and KD (golden) mice based on their branch order and whether they arise from the PA or AV. **E** Timeline of the rPAI-1 in vivo experiment. **F** Example z-projection image of a vessel dilating post-rPAI-1 treatment. Scale bar = 20 µm. **G** Graph showing vessel width in *Serpine1* WT mice across the imaging time points. **H** Graphs showing percent change of vessel width at 6 and 24 h when normalized to baseline (-7 days, pre-treatment; %) of individual vessels (left) and mean (right). **I** RBC velocity measurements in capillaries across the imaging time points. Data in (**C**, **D**) were analysed with two-way ANOVA followed by Tukey’s multiple comparisons test. Comparisons between *Serpine1* KD vs WT: **** *p* < 0.0001, *** *p* < 0.001, ** *p* < 0.01, * *p* < 0.05. Comparisons between post-stroke time-point to their respective pre-stroke value: #### *p* < 0.0001, ### *p* < 0.001, ## *p* < 0.01, # *p* < 0.05. *N* = 6 sham stroke mice, *N* = 10 and 17 stroke-affected *Serpine1* WT and KD mice, respectively. Data in **G**, **I** were analysed with one-way ANOVA followed by Tukey’s multiple comparisons test. Comparisons between time of rPAI-1 treatment: * *p* < 0.05. Data in **H** was analysed with a paired t-test comparing to pre-treatment, and a one-sample t-test compared to pre-treatment as a hypothetical of zero. Comparisons between time of rPAI-1 treatment: ** *p* < 0.01. *N* = 4 *Serpine1* WT mice, *n* = 56 vessels for (**G**, **H**) and *n* = 12 vessels for (**I**). Data expressed as the mean ± SD.

Given the reduction in RBC velocity/flux, we measured stroke-induced changes in the widths of capillaries from the 1st to 6th order capillary branch off a PA or AV (Fig. 3A, B). As expected [37], capillary widths decreased with increasing branch orders (Fig. 3C, D). Consistent with our RBC velocity and flux measurements, we noted a transient dilation of 1st–3rd order capillaries 3 days after stroke in WT mice (see blue hashtags in Fig. 3C). Conversely, *Serpine1* KD mice showed a highly significant and persistent reduction in capillary widths after stroke that was most evident in capillaries arising from the PA (Fig. 3C). For more distant regions, similar trends were observed although the magnitude of stroke and genotype-based differences were generally reduced (Fig. 3D). To confirm the potential dilatory effects of PAI-1, we imaged vessel width and RBC velocity in *Serpine1* WT mice before and after (Pre, +6 and 24 h) application of recombinant PAI-1 to the cortex (Fig. 3E, F). We detected a significant increase in absolute and normalized vessel width at 6 h post-treatment but not at 24 h (Fig. 3G, H). In addition, blood flow was also significantly increased at 6 h only (Fig. 3I). To conclude, *Serpine1* KD mice exhibit a pronounced stroke-related constriction of capillaries, while stimulating PAI-1 signalling leads to a moderate increase in capillary width and flow.

### Endothelial *Serpine1* knockdown reduces BBB permeability after stroke

**Fig. 4:**
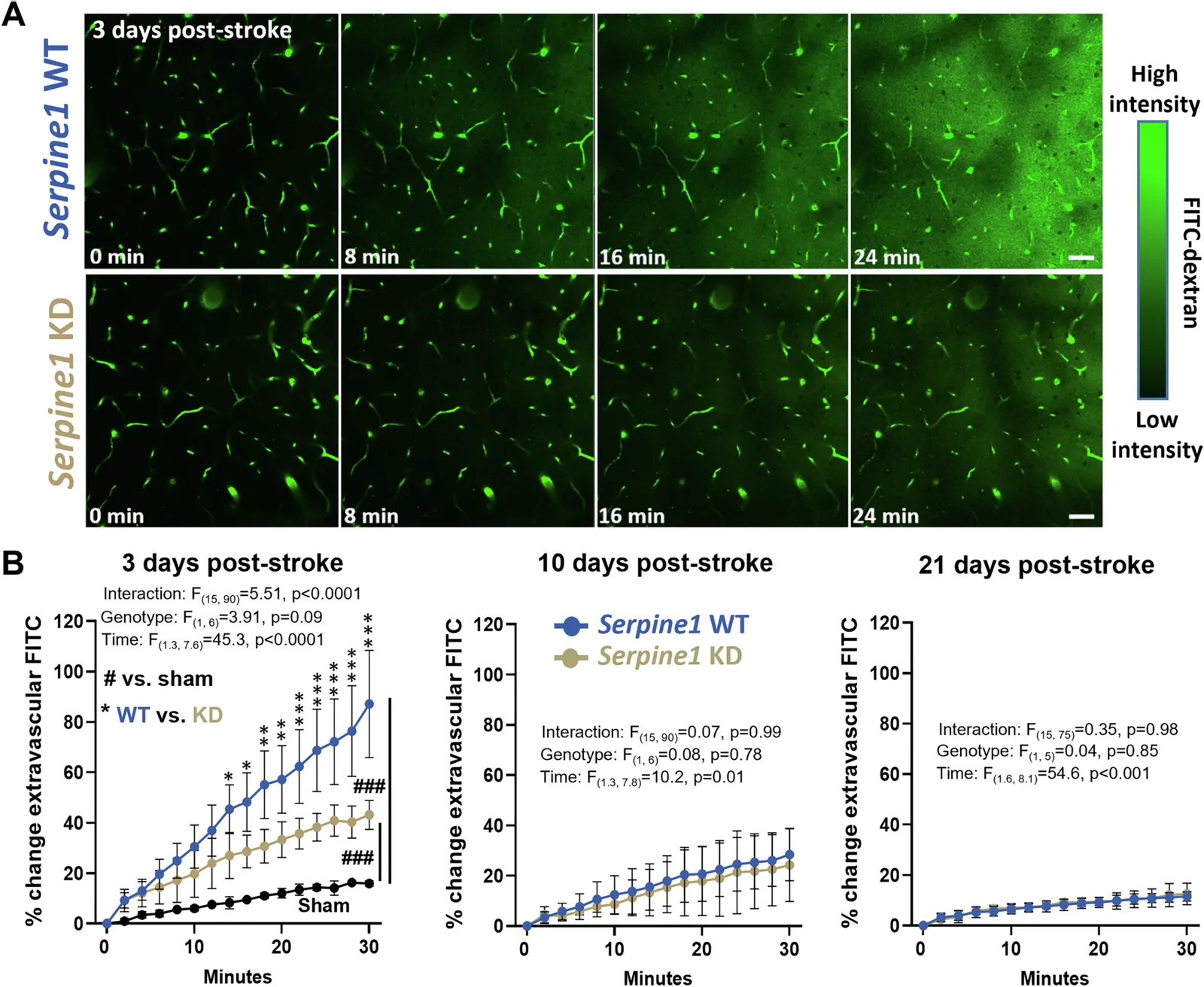
*Serpine1* KD reduces blood-brain barrier permeability in peri-infarct cortex. **A** In vivo maximum intensity z-projection images showing FITC-dextran extravasation in peri-infarct cortex of *Serpine1* WT and KD mice at 3 days post-stroke (*n* = 6 mice per group). Scale bar = 50 µm. **B** Graphs show the percent change in extravascular FITC in *Serpine1* WT and KD mice (*n* = 6 mice/group) at 3, 10 and 21 days post-stroke. Sham stroke controls are plotted in black for comparison (*n* = 4 mice based on 2 WT and 2 KD). Data were analysed with two-way ANOVA followed by Tukey’s multiple comparisons test. Comparisons between *Serpine1* KD vs WT: *** *p* < 0.001, ** *p* < 0.01, * *p* < 0.05. Comparisons to sham stroke controls: ### *p* < 0.001. Data expressed as the mean ± SD.

A reduction in peri-infarct blood flow velocity and flux after stroke could be beneficial for recovery if it were to reduce BBB breakdown, which is well known to have deleterious effects on local synaptic circuits and stroke recovery [38]. To assess permeability of the BBB in vivo, we quantified the extravasation of FITC-dextran (4 kDa) labelled blood plasma in peri-infarct cortex (Fig. 4A) [39, 40]. At 3 days post stroke, BBB permeability was significantly reduced in *Serpine1* KD mice compared to WT mice (Fig. 4B). Imaging at later recovery time points (10 and 21 days) did not reveal any differences based on genotype (Fig. 4B), which is consistent with previous data showing repair and normalization of BBB integrity over time [38]. And finally, it is important to note that we did not find evidence of larger-scale vascular disruption in the form of haemorrhages in peri-infarct cortex after *Serpine1* knockdown.

Inducing photothrombosis across the entire region of the FL cortex (as we have done in this study) does not yield much of a penumbra nor mimic ischemic stroke in humans that involves the occlusion of an artery. Therefore, we assessed BBB permeability and infarct volumes in post-mortem tissue 3 days after photo-thrombotically occluding a distal branch of the MCA [41]. Consistent with our findings, *Serpine1* KD mice exhibited significantly reduced Evans blue extravasation compared to WT mice within 200 µm from the infarct border (Supplementary Fig. 3A, B), which represents the same area defined as “peri-infarct” from in vivo imaging. Furthermore, there was no difference in infarct volume between genotypes (Supplementary Fig. 3C). Together, we conclude that BBB permeability is substantially reduced in *Serpine1* KD mice following stroke.

### Endothelial *Serpine1* knockdown alters inflammation-related gene and protein expression

**Fig. 5:**
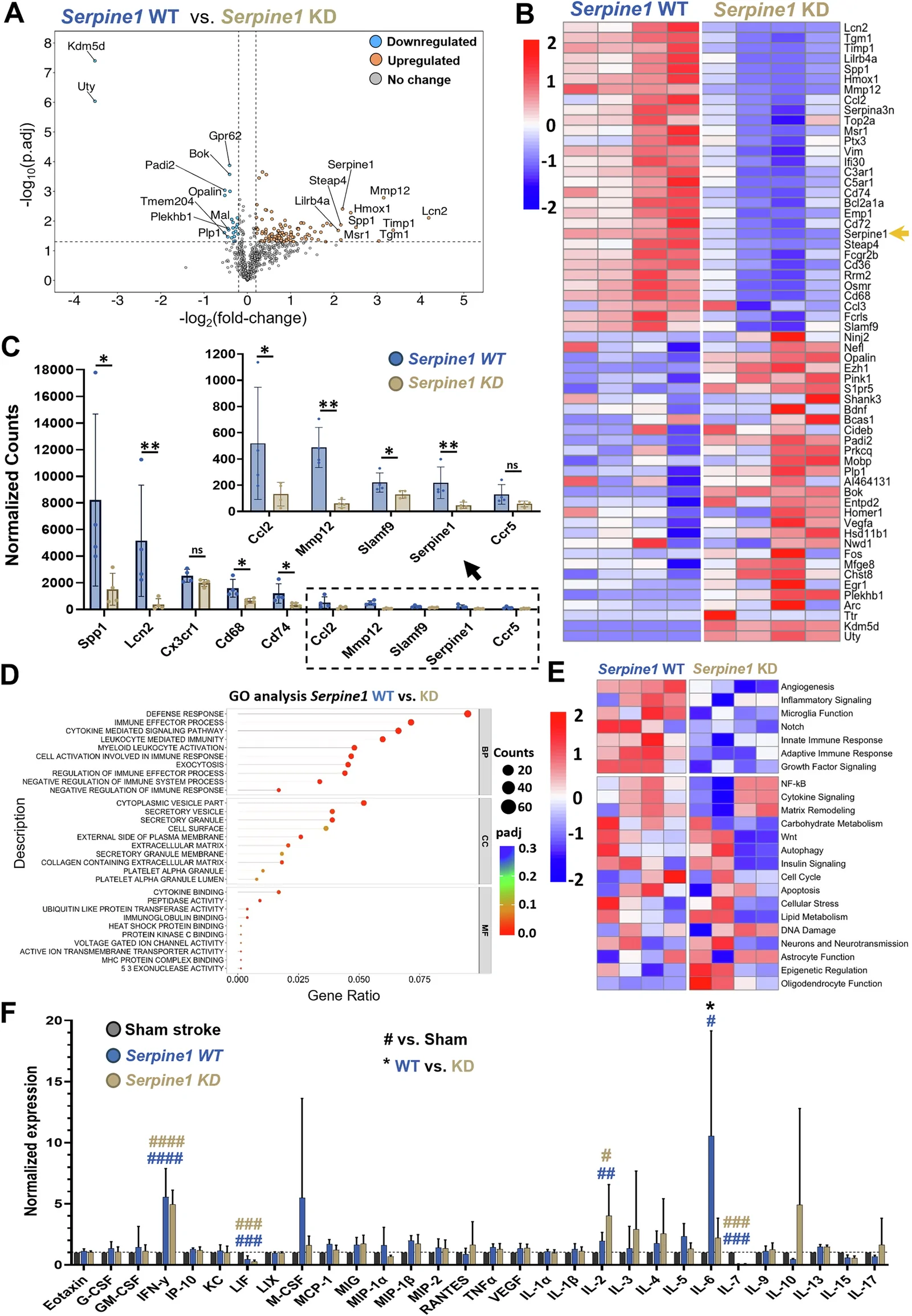
*Serpine1* KD alters inflammatory gene and cytokine expression after stroke. **A** Volcano plot of 770 differentially expressed genes (DEGs) in peri-infarct tissue of *Serpine1* WT versus KD normalized to sham stroke (*n* = 4 mice/group), with the top 10 most up- (orange) and down-regulated (blue) labelled. Data is analysed with a moderated t-test with multiple test corrections using limma with false discovery rate (FDR) < 0.05, and an absolute value of log_2_ FC > 0.3 is coloured as dotted lines. **B** Heatmap showing log_2_ fold change (FC) of the top-30 most significantly up- and down-regulated genes in both genotypes (WT-left, KD-right). The scale for relative gene expression changes ranges from +2 (up-regulated, red) to -2 (down-regulated, blue). **C** Histogram shows normalized gene counts of the top-10 most significantly regulated inflammatory genes (*p*_*adj*_ < 0.05 for all com*p*arisons between genotypes). Data expressed as the mean ± SD. **D** Dot plot showing GO enrichment analysis of DEGs (two-sided test) for the most significantly regulated processes in *Serpine1* WT vs KD; BP – biological process, CC – cellular component, and MF – molecular function. **E** Heatmap of log_2_ fold change of the most regulated pathways in both genotypes. The top 7 most differentially regulated processes/pathways between groups are shown in the top panel. DEGs in **B**, **E** with FDR < 0.05 were ranked by their log_2_FC, and z-scores were computed on average gene expression for visualization in heatmaps. **F** Cytokine expression in blood serum at 3 d post-stroke, normalized to sham control (*n* = 4 mice/group). One-sample t-tests were used to compare stroke to sham, while an unpaired two-tailed t-test was used to compare *Serpine1* WT and KD. Comparisons between *Serpine1* KD vs WT: **** *p* < 0.0001, *** *p* < 0.001, ** *p* < 0.01. Comparisons between each genotype and sham stroke control: #### *p* < 0.0001, ### *p* < 0.001, ## *p* < 0.01.

We next asked whether reduced BBB permeability after stroke could be associated with inflammation. First, we characterized differentially expressed genes (DEGs) in *Serpine1* WT and KD in peri-infarct tissue at 3 days post-stroke (*n* = 4 mice/group, see Fig. 2B for experimental summary) and compared them to sham controls consisting of both *Serpine1* WT and KD mice (*n* = 4 total with 2 mice per genotype). As expected, several injury/inflammation-related genes were significantly upregulated after stroke in *Serpine1* WT and KS mice when compared to sham controls (Supplementary Fig. 4A–D), including *Serpine1* (in WT only)*, Serpina3n, Spp1, Cd68, Cd74, Slamf9, Lcn2, Ccl2, Mmp12* and *Timp1*. Directly comparing *Serpine1* WT vs. KD mice at 3 days post-stroke showed that many of these injury/inflammation-related genes were significantly decreased in *Serpine1* KD mice (Fig. 5A–C). Gene ontology also revealed that inflammation related gene families were more strongly expressed in *Serpine1* WT than KD mice (Fig. 5D). Similarly, KEGG analysis showed that gene functions most strongly down-regulated in *Serpine1* KD mice were associated with microglia function, innate and adaptive immune responses, as well as Notch and growth factor signalling (Fig. 5E). In addition to genomic alterations, we also analysed cytokine levels in blood serum collected 3 days after stroke or sham stroke procedure. When examining stroke-related differences between genotypes (see asterisks in Fig. 5F), *Serpine1* KD mice had significantly lower levels of IL-6 relative to *Serpine1* WT mice. These results suggest that *Serpine1* knockdown leads to a reduction in inflammation-related gene expression.

Given these changes, we next examined the extent of neutrophil invasion in peri-infarct and core regions at 3 days after stroke (Supplementary Fig. 5A). Our analysis of Ly6G immunolabelled neutrophils did not reveal any significant differences in either region based on genotype (Supplementary Fig. 5B). Thus, *Serpine1* KD does not appear to alter the recruitment of neutrophils to stroke-affected regions.

### Endothelial *Serpine1* knockdown promotes recovery of somatosensory cortical function

**Fig. 6:**
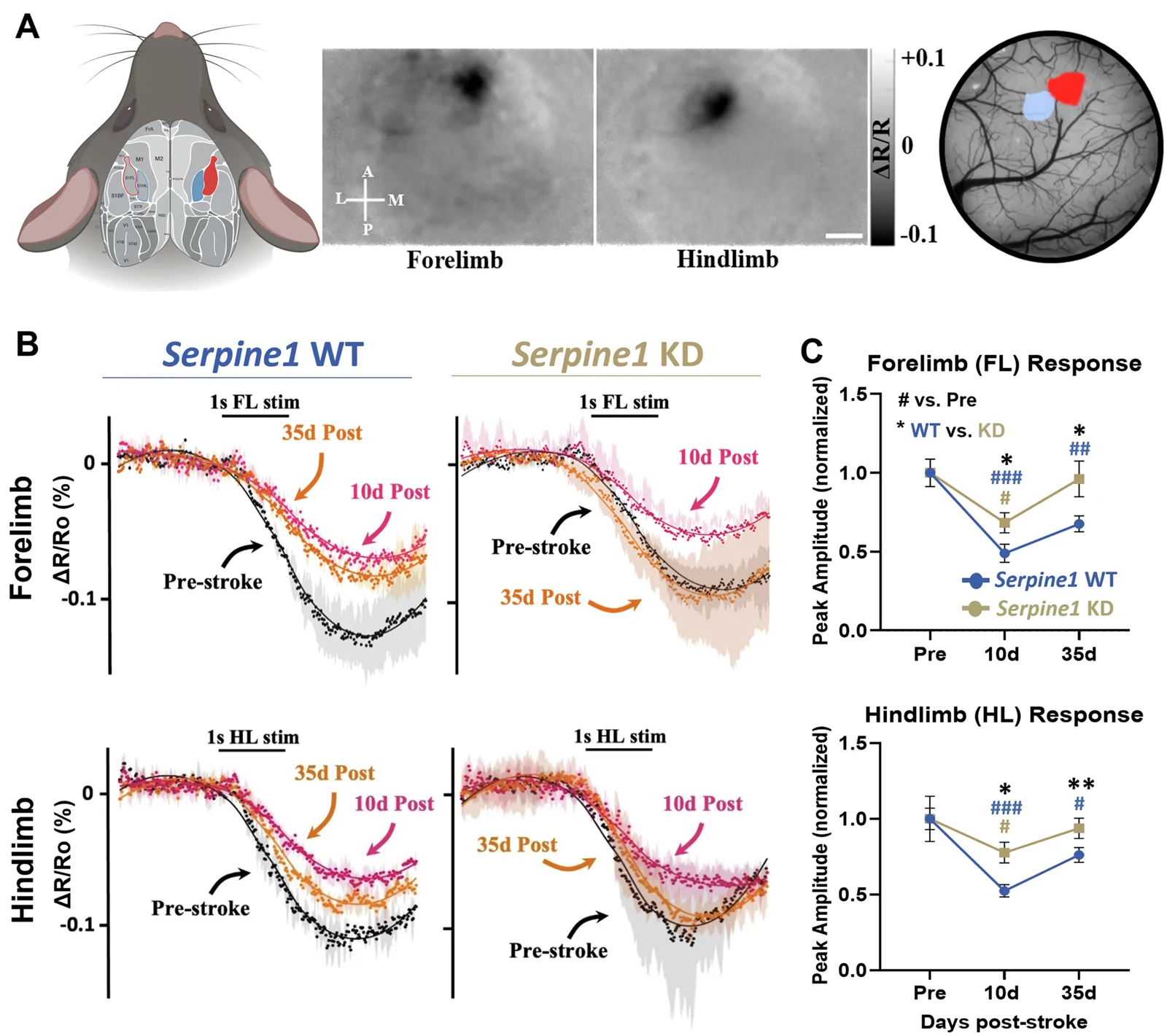
*Serpine1* KD mice show enhanced recovery of sensory evoked cortical responses after stroke. **A** Left: Mouse brain shows a map of forelimb (FL, red) and hindlimb (HL, blue) regions. Middle: representative FL and HL evoked IOS signals based on changes in light reflectance relative to pre-stimulus baseline (% ΔR/Ro). Each response was thresholded and superimposed onto the cortical surface image. Scale bar = 1 mm. **B** Graphs show average change (% ΔR/Ro) of the FL, and HL evoked cortical response in *Serpine1* WT (left, blue, *n* = 7 mice) and *Serpine1* KD (right, golden, *n* = 10 mice), measured pre-stroke (black), 10 d (pink) and 35 d (orange) post-stroke. **C** Graphs show peak amplitude of FL and HL evoked responses in each genotype, normalized to pre-stroke values at 10 days and 35 days after stroke. Data in **C** analysed with two-way ANOVA and Tukey’s multiple comparisons test. Comparisons between *Serpine1* KD vs WT: ** *p* < 0.01, * *p* < 0.05. Comparisons between each genotype and Pre-stroke value: ### *p* < 0.001, ## *p* < 0.01, # *p* < 0.05. Data expressed as the mean ± SD.

We next probed functional indicators of stroke recovery in the longer term (see summary diagram in Fig. 1B). For example, recovery of cortical responses in the stroke-affected hemisphere is predictive of behavioural recovery [42–44]. Thus, we imaged sensory-evoked intrinsic optical signals (IOS) in *Serpine1* WT and KD mice. IOS signals reflect local increases in deoxyhaemoglobin levels due to an increase in oxygen demand from active neurons, providing an estimate of functional cortical activity after stroke. However since IOS signals are vascular in origin and can be influenced by systemic changes in cardiovascular function, we examined oxygen saturation, heart rate and breath rate and found no significant differences between genotypes (Supplementary Fig. 6). We also analysed blood chemistry, which did not reveal any genotypic differences in haematocrit and haemoglobin, although there were some subtle group differences in Na^+^ and HCO_3_ levels which were still within the normal range (Supplementary Fig. 7). In the absence of stroke, fore- or hindlimb (FL and HL, respectively) stimulation elicits a robust response in primary somatosensory cortex (Fig. 6A). The amplitude of sensory evoked responses before stroke did not differ between genotypes for both FL (*F*_(1, 2617)_ = 13.21, *p* > 0.99) and HL cortex (*F*_(1, 4352)_ = 10.54, *p* > 0.99). At 10 days post-stroke, FL and HL cortical responses were significantly reduced in amplitude in both genotypes, albeit to a lesser degree in *Serpine1* KD mice relative to WT (Fig. 6B, C). By 35 days of recovery, *Serpine1* KD IOS responses had recovered back to pre-stroke levels, whereas *Serpine1* WT responses remained significantly below baseline levels (Fig. 6B, C). These results indicate that *Serpine1* KD helps preserve and/or recover somatosensory cortical function after stroke.

### *Serpine1* knockdown improves behavioural recovery from ischaemic stroke

**Fig. 7:**
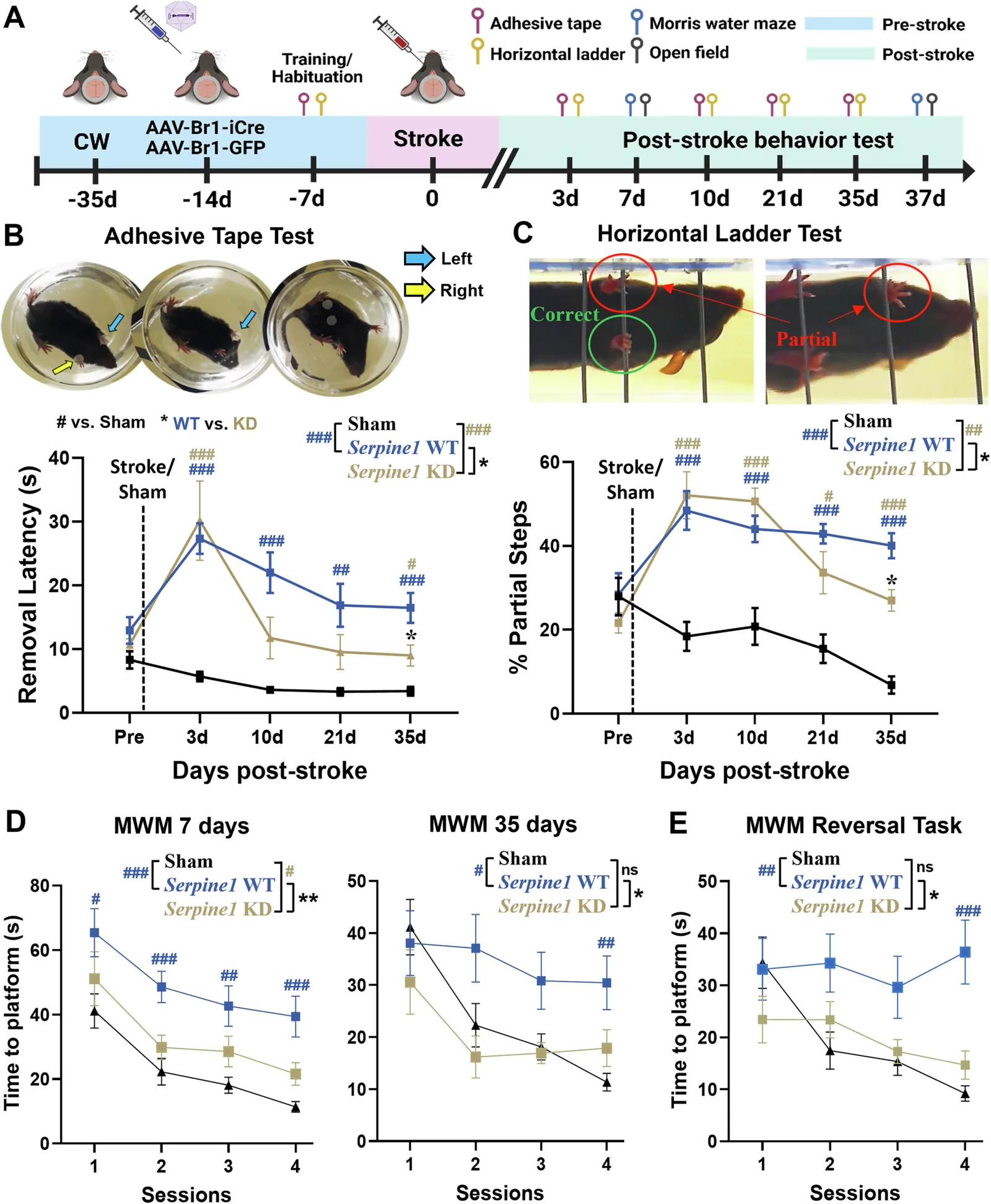
*Serpine1* KD mice show improved recovery of sensori-motor and cognitive function. **A** Experimental timeline for behavioural tests. **B** Representative example of a stroke- affected mouse removing adhesive tape from left and right paws. Graph shows latency of tape removal (s) in the three groups: sham stroke (black, *n* = 15 mice), *Serpine1* WT (blue, *n* = 13 mice) and *Serpine1* KD (golden, *n* = 16 mice). **C** Representative example of a stroke-affected mouse crossing the horizontal ladder test, circles denote correct and partial paw placements. The graph shows the average number of partial placement steps as a percent (%) of total steps. **D** Escape latency for learning the hidden platform location in Morris water maze (MWM) at 7 days (left) and 35 days (right) post-stroke. **E** Average escape latency for learning new platform location in MWM (‘reversal task’). Data in **B**–**E** analysed with two-way ANOVA and Tukey’s multiple comparison tests. Comparisons between *Serpine1* KD vs WT: ** *p* < 0.01, * *p* < 0.05. Comparisons between each genotype and Pre-stroke or Sham stroke value: ### *p* < 0.001, ## *p* < 0.01, #*p* < 0.05. Data expressed as the mean ± SD.

Next, we subjected mice to behavioural tests to determine if *Serpine1* KD ultimately improved function after stroke (Fig. 7A). For the tape removal test, *Serpine1* KD mice exhibited significantly reduced tape removal latencies from day 10 onwards (from the stroke affected left paw) compared to *Serpine1* WT mice (Fig. 7B). In the horizontal ladder test, stroke led to a significant increase in the percentage of partial/incorrect forepaw placements in both *Serpine1* WT and KD mice when compared to sham controls (Fig. 7C). At later time points, *Serpine1* KD exhibited marginally better performance, which was significant at 35 days (Fig. 7C). To assess spatial learning and memory, we tested mice in the Morris water maze (MWM) at 7- and 35-days post-stroke (Fig. 7D). *Serpine1* KD mice performed significantly better than *Serpine1* WT mice at both 7 days (Fig. 7D; 2-way ANOVA, Genotype: *F*_(1, 51)_ = 10.12, *p* = 0.002) and 35 days recovery (2-way ANOVA, Genotype: *F*_(1, 49)_ = 6.48, *p* = 0.01). To assess cognitive flexibility (i.e., “reversal learning”). *Serpine1* KD mice took significantly less time to find the new submerged platform location compared to *Serpine1* WT mice (Fig. 7E, 2-way ANOVA, Genotype: *F*_(1, 50)_ = 5.66, *p* = 0.02). And finally, we tested whether there were any general changes in locomotor activity and exploration, and did not find any differences based on genotype (Supplementary Fig. 8). In summary, our data show that *Serpine1* KD mice display improved sensori-motor and cognitive function after stroke.

## Discussion

Optimal recovery from stroke is dependent on proper macro- and microvascular blood flow [45–47]. In the first few h after an ischaemic event, this undoubtedly means restoring adequate blood flow to allow vulnerable cells to survive [48–50]. At the microvascular level, preventing the stalling of peri-infarct capillaries in the first few h after stroke, even after recanalizing large vessels with tPA, enhances the return of cerebral blood flow in capillaries, reduces ischaemic infarct volume, and improves outcome [51, 52]. Over the longer term (days and weeks), what constitutes “optimal” blood flow, particularly at the capillary level, is unclear. Conventional logic might suggest that increased flow in peri-infarct regions would be better for recovery. Indeed, this is what motivated us to inhibit *Serpine1*/PAI-1 signalling in the present study. However, as we discovered, enhanced functional recovery in *Serpine1* knockdown mice was associated with a reduction in capillary flow. Modelling studies suggest that chronic hyperaemia (increased flow) could disturb capillary flow heterogeneity, reducing oxygen extraction [53, 54]. It is important to note that enhanced recovery in *Serpine1* knockdown mice could not be explained by an acute neuroprotective effect (i.e., salvaging more penumbra) since blood flow reductions at 6 h and infarct volumes were similar between groups. This finding differs slightly from previous studies showing that systemic inhibition of PAI-1 or congenital knockout of *Serpine1* improves stroke outcome by reducing the volume of infarction [26, 55, 56]. However, neither previous study explored cell-specific knockdown/inhibition approaches nor examined functional recovery beyond 24 h. Thus, our study adds important new data regarding endothelial-specific manipulations of *Serpine1*/PAI-1 signalling and its long-term effects on stroke recovery.

If *Serpine1* knockdown does not provide early neuroprotection, at least in our model, then how might it lead to better recovery? Since infarct volume is often considered a weak predictor of long-term functional recovery [57, 58], perhaps BBB disruption is a critical factor [38, 59, 60]. Stroke disrupts the BBB for multiple days, which perturbs neuronal structure and function in surviving peri-infarct regions and worsens stroke outcome [61, 62]. Previously, we showed that increased BBB permeability and poor functional outcome in stroke-affected diabetic mice were accompanied by abnormally increased peri-infarct capillary flow [38, 45]. The idea that elevated blood flow in peri-infarct capillaries leads to increased BBB permeability (or vice versa) is plausible since these capillaries are in a vulnerable state with heightened remodelling, and increased expression of ion channels and growth factors that modulate blood flow and BBB integrity [63–68]. Indeed, we found that peri-infarct BBB permeability was lower in *Serpine1* KD mice at 3 days of recovery. The reduction in BBB permeability in *Serpine1* KD mice could have lessened neuro-inflammation and potentially damaging processes associated with it. Transcriptomic and cytokine expression analysis support this idea since *Serpine1* KD mice showed lowered expression of putative proinflammatory genes (e.g., *Spp1, Cd74, Slamf9, Ccl2, Mmp12*) and cytokines (IL-6). Our findings are reminiscent of work showing that returning blood flow too quickly in the hyper-acute stage of stroke ( < 24 h) with too few collaterals leads to haemorrhages and worse stroke outcome in mice and humans [4, 69]. In summary, our data suggest that a reduction in peri-infarct capillary blood flow could be beneficial for stroke recovery by attenuating BBB disruption and neuroinflammation.

While *Serpine1*/PAI-1 signalling is best known for its role in inhibiting fibrinolysis, and found supportive evidence of that here (see Supplementary Fig. 1), there are reports that it can modulate vessel tone directly or indirectly [18]. Our study is the first to show that endothelial *Serpine1* KD leads to constriction of peri-infarct capillaries. Consistent with an effect on tone, capillaries branching off penetrating arterioles, which are enriched in smooth muscle cells or pericytes [35, 36, 70] showed greatest constriction. We also demonstrated that cortical application of PAI-1 increased capillary flow and diameter, which could explain why there is a transient increase in vessel diameter in WT mice at 3 days, replicating previous data [45]. We should note that our study focused mostly on resting blood flow. However, when evoking a putative change in cortical activity and blood flow, *Serpine1* KD mice did show enhanced sensory evoked IOS signals, agreeing with work showing enhanced sensory-evoked blood flow responses following systemic inhibition of PAI-1 [71, 72]. Larger sensory evoked IOS or blood flow responses with PAI-1 inhibition/knockout could reflect a greater dynamic range of response because narrower vessels (at rest) would allow for greater increases in blood flow when stimulated. While the signalling mechanisms that couple PAI-1 with changes in vessel tone remain unclear, PAI-1 is highly enriched in myo-endothelial junctions [73] and can interact with eNOS signalling [74, 75]. Further, PAI-1 can dose dependently stimulate arterial contractility in the heart [18]. Undoubtedly, more work will be needed to resolve precisely how PAI-1 influences vessel tone.

While our study provides novel pre-clinical evidence implicating endothelial *Serpine1*/PAI-1 signalling as a determinant of stroke recovery, there are limitations. First, given the technical challenges that are inherent with long-term in vivo imaging, our study was not sufficiently powered to detect sex differences. Resolving potential sex differences is a critical issue [76] but would require many more samples and thus be better served in a future study. Second, our experiments were conducted in lightly anesthetized mice, which could influence our findings; future studies could examine vascular changes in awake mice. Third, our analyses provide only an indirect approximation of CBF rather than absolute CBF measurements. Although the observed reductions in peri-infarct flow were not associated with infarct expansion, impaired cortical responsiveness, or worsened functional outcomes, future studies incorporating quantitative volumetric measurements of CBF and imaging of neural structure would help further define when reductions in peri-infarct perfusion become detrimental. Lastly, the translational significance of our study could be questioned since our cell-specific knockdown was initiated before, not after, stroke. However, we are encouraged by new drug delivery systems that target endothelial cells [77] and could, in the future, be initiated after stroke. These cell-specific manipulations are an important consideration for future studies since they could avoid unwanted side effects such as haemorrhages that are usually associated with systemic manipulations of fibrinolysis. In this light, our study raises the possibility that endothelial inhibition of *Serpine1*/PAI-1 can improve stroke recovery without inducing vascular complications.

## Supplementary information

Supplementary data

## Data Availability

All data and code generated for the study are available from the corresponding author upon reasonable request. Transcriptomic datasets generated during and/or analysed during the current study are available in the NCBI Gene Expression Omnibus (GEO) repository, accession number: GSE331241
