# Supplementary material for: Knockdown of endothelial Serpine1 improves stroke recovery by attenuating peri-infarct blood flow and blood-brain barrier disruption
Source: Cell Death Dis. 2026 Jun 27;17(1):800. doi: 10.1038/s41419-026-09062-9 (PMC13575155; doi:10.1038/s41419-026-09062-9)
Supplement: Supplementary file 1 [file 41419_2026_9062_MOESM1_ESM.pdf]

Supplementary data for:

Knockdown of endothelial *Serpine1* improves stroke recovery by attenuating peri-infarct blood flow and blood brain barrier disruption

Kamal Narayana[1], Isabel C. Lambert[1], Sam Burford[1], Emilie Gosselin[1], Jakob Körbelin[3], and Craig E. Brown[1,2]*

Supplemental data consist of:

i) Supplemental methods and references
ii) Supplemental Figures 1-8
iii) Supplemental Table 1

Supplemental Methods

Cranial window preparation

Mice were anaesthetized with isofluorane (2% induction and 1.2% maintenance) in medical grade air at a flow rate of 0.7 L/min and head-fixed to a custom-built surgical stage. Animal's body temperature was maintained at 37°C throughout the procedure. Lidocaine was injected subcutaneously under the scalp at the surgical site, and 0.03mL bolus of 2% dexamethasone was injected intramuscularly to reduce inflammation. A midline incision was performed followed by scalp retraction. After teasing off soft tissues on the skull, a custom metal ring (outer diameter 11.3mm, inner diameter 7.0mm, height 1.5mm) was affixed to the skull with Metabond adhesive over the right somatosensory cortex (~2mm right of bregma). Using a high-speed dental drill, two circular areas were thinned overlying the somatosensory cortex: the outer ring 6mm in diameter, and the inner ring 5mm in diameter. Cold (<4°C) HEPES-buffered artificial cerebrospinal fluid (ACSF) was applied periodically throughout to cool the exposed brain surface and reduce inflammation. The inner area of the skull was removed with forceps leaving the dura intact and cold ACSF was left on the brain surface for 15min to reduce possible swelling. A custom glued cover slip (6mm outer, 5mm inner) was placed over the intact dura and secured to the skull with cyanoacrylate glue and dental cement. Mice were allowed recover on a heating pad before being returned to their cage.

IOS imaging and pulse oximetry

Intrinsic optical signal imaging (IOS) was used to non-invasively and indirectly examine neural activity in primary somatosensory cortex. Mice with cranial windows previously installed

were lightly anaesthetized with ~1% isofluorane in medical air and fitted to a custom imaging stage. A pencil lead (0.7mm) connected to a piezoelectric bending actuator (Piezo Systems, Q220-A4-203YB; 300µm deflection) was lightly glued to the FL and HL separately. The cortical surface was illuminated with a red LED (625nm) coupled to a collimation lens. Sensory-evoked changes in reflected light were collected for FL and HL (separately) using the MiCAM-2 CCD camera (SciMedia) through a 2X objective lens. Each imaging trial was collected over 3s at 100Hz with a 10ms exposure. Vibrotactile stimulation (100Hz) for each limb was initiated after 1s in each 3s trial and lasted for 1s. Two sets of 12 sensory stimulation trials were recorded for each limb. Using FIJI software, the 24 trials were averaged and then mean filtered (radius 5 pixels). Each image in the averaged and smoothed image stack was then subtracted and divided from a mean intensity projection of the pre-stimulus period (frames from 0-1s). Delineation of FL and HL regions was based on thresholding an average intensity image projection (projected frames from 0.5-2s after stimulation) at half-maximal intensity. IOS signals were then measured by placing an ROI (0.5mm diameter) in the centre of the IOS response. Peak amplitudes for FL and HL responses were then measured using Clampfit software.

Physiological parameters such as O_2 saturation, heart and breath rate were measured during IOS imaging sessions before stroke and 3 days post-stroke. A cuff was placed onto the mouse hindlimb and measurements were recorded at 10Hz using the MouseOx small animal vital signs monitor (Starr Life Sciences) for a total of 15-20min at each time point.

Histology

Mice were administered an overdose of sodium pentobarbital. Brains were extracted and then either frozen in dry ice or fixed overnight in 4% PFA followed by 30% sucrose solution in 0.1M PBS with 0.2% sodium azide. Fresh frozen brains were sectioned on a cryostat at 40µm and fixed in ice cold methanol, whereas PFA fixed brains were sectioned at 40µm on a freezing microtome (American Optical Corp.) and stored in 0.1M PBS with 0.02% sodium azide solution. For antigen retrieval in PFA fixed brains (Note: this is not needed for fresh frozen tissue), sections were incubated in sodium citrate buffer (10mM, pH = 6.0) for 30min at 75°C. Following a wash in 0.1M PBS for 5 mins, sections were incubated with a monoclonal PAI-1 primary antibody (1:1000, Invitrogen #MA5-17171) and polyclonal goat anti-Collagen 4 (1:100, Millipore, AB769) overnight at 4°C. Sections were washed with PBS followed by incubation with secondary antibodies to mouse and goat (Cy5 or Alexa Fluor 488 conjugated antibodies; 1:500, Invitrogen) for 4 hrs at room temperature. Sections were mounted, coverslipped and imaged using an Olympus confocal microscope at 10x (NA = 0.40) and 20x objective (NA = 0.75).

Fluorophores Cy5 and/or 568 were excited at 635 and 568nm lasers, respectively. Image stacks were collected with a Kalman filter (average = 2 frames) in 2 (for 10x obj. lens) or 1 (for 20x obj. lens) µm Z-steps with pixel sampling set at 1.24 and 0.621µm/pixel, respectively. For vessel analysis, stacks were maximally z-projected and automatically thresholded using built in ImageJ/Fiji threshold function Triangle[1], which best identified vessel signal. A median filter (radius 1 pixel) was applied to eliminate speckling artifact, and the remaining area of vascular signal was measured within the peri-infarct region, and then normalized to sham stroke controls.

For labelling of neutrophils 3 days after stroke, 40µm thick PFA fixed brain sections were incubated in rat anti-Ly6G primary antibody (1:1000; Biolegend, clone 1A8, Cat#127602) for 2 days at 4°C. Sections were washed in PBS and then incubated in Cy5 conjugated goat anti-rat secondary antibody (1:500 in 0.1M PBS) at room temperature for 4 hours. Sections were washed once in PBS, incubated in Hoescht 33342 (1:1000 Invitrogen Cat#H3570) for 10min, washed again and then mounted onto slides and coverslipped. Confocal images were collected with a 10X objective (NA = 0.40) using 405, 488 and 635nm laser lines with corresponding emission filters (Alexa 405, 488, 647) to sequentially image cell nuclei, stroke related autofluorescence and Ly6G labelled neutrophils, respectively. Image stacks were collected with a Kalman filter (average = 2 frames) in 2µm Z-steps with pixel sampling set at 0.8µm/pixel. For determining the stroke border, an average image projection of the stroke related autofluorescence (via the Alexa488 channel) was generated and then mean filtered (20 pixel radius). The autofluorescence defined infarct border was verified by comparing with images of cell nuclei. For quantifying Ly6G labelled neutrophils, a maximum intensity projection image was first generated. We then subtracted out the background fluorescent signal by median filtering the original image with a 50 pixel radius. The resulting image of Ly6G cells was then binarized with an Otsu threshold. Using the “Analyse Particles” function in ImageJ/Fiji, we quantified the number of Ly6G labelled cells within the peri-infarct region (0-200µm outside the infarct border) and within the infarct core (0-200µm within the infarct border). Neutrophil cell number was normalized to volume of tissue sampled.

Behavioural testing

Adhesive tape removal and horizontal ladder tests were included for their high sensitivity in detecting damage-related changes in sensory-motor function of the forepaw[2–4]. To assess spatial learning and memory we performed Morris Water Maze[5] as well as a reversal learning test for cognitive flexibility[6]. And to assess locomotive ability, we performed open-field test [7]. Behavioural tests were administered 2 weeks before stroke and at each two-photon imaging

time-point afterwards; behavioural tests were conducted in the morning, and imaging in the late afternoon. All of the following tests are described in our previous studies [3,8–10] and were scored blind to condition.

Tape Removal Test. A circular piece of tape (5mm diameter) was adhered to the palmar surface of each forepaw. Mice were then placed in a clear glass cylinder and filmed from below for up to 120s until the tape pieces were removed from both forepaws. The latency to remove the tape from each forepaw was recorded in seconds and both forepaws were averaged as tape removal latency (s). This was repeated three times per testing session.

Horizontal Ladder Test. Mice were videotaped walking across an elevated 70cm long horizontal ladder that had 1mm diameter rungs randomly spaced 1 or 2cm apart. Grasping of the rungs by the forepaws were scored on a frame-by-frame basis based on the centrality and completeness of the forepaw placement. The scores were assigned by a blind observer as follows: (i) “correct” (forepaw placement centred), (ii) “partial” (forepaw placement partially centred), (iii) “slip/miss” (forepaw placement not centred).

Open field test. Mice were placed into the centre of a 100cm diameter circular arena, and the total distance travelled (in cm) was automatically recorded and measured using Ethovision software (Noldus Information Technology, Version 11.5.1020, 2015). One trial of 7min was conducted for each mouse 2 weeks pre-stroke, and 7 and 35 days post-stroke.

Morris Water Maze. In a large circular water-filled pool (100cm diameter), a hidden platform was placed approximately 1cm below the surface in one of four quadrants. Non-toxic white paint was added to the water to hide the platform, and water temperature was maintained at room temp. Mice were trained to locate the hidden platform using unique shaped-visual cues (maintained throughout experiment) placed around the maze. Mice were then placed in one quadrant facing a specified starting point: northeast (NE), southeast (SE), northwest (NW), or northeast (NE). The trial ended when the platform was located and mice stopped on the platform for three seconds, or until a maximum of two minutes had elapsed without the platform being located. The training sessions were performed over 2 days for a total of four training sessions. To score escape latency (seconds) and swim speeds (cm/s) to platform, the Ethovision software was used. For the reversal task, we switched the platform to a new location and performed the test in the same manner as the initial training and trial sessions.

NanoString and cytokine assays

In brief, mice were deeply anaesthetized and perfused with cold, sterile 0.1M PBS for 30s before tissue extraction. Peri-infarct regions in stroke mice or equivalent regions in sham

controls (n = 4 per group) were isolated and flash-frozen in liquid nitrogen and stored at -80°C. A minimum of approximately 100ng of total RNA was used, and RNA quality and quantity (A_{260}/A_{280}) were estimated using Nanodrop 200 spectrophotometer (ThermoFisher, ND-2000). Samples were processed using the NanoString nCounter mouse neuroinflammation panel (770 genes + 6 internal reference controls; Catalogue IDL XT_Mm_Neuroinflam_CSO – 115000237, Nanostring Technologies, Seattle, WA, USA). Hybridization was performed using nCounter Master Kit (Catalogue # NAA-AKIT-012, Bruker). The number of occurrences of the hybridized cDNA to the unique bar-carded probes was counted by Nanostring nCounter instrument and raw gene counts were obtained for further analysis. Nanostring nSolver version 4.0 was used to extract raw gene counts for all tissue samples. All experimental samples were organized based on their condition; Sham stroke, *Serpine1* WT stroke, and *Serpine1* KD stroke. Background threshold was calculated as 12 counts, using the 'mean negative probe counts + 2 [standard deviation of negative probe counts]' formula, and gene counts < 12 were excluded from the analysis. Gene counts were then normalized to positive control and housekeeping probes, and resulting normalized and annotated data was used for subsequent analysis.

For examining cytokine levels in blood serum, mice were deeply anaesthetized and blood was collected and allowed to clot at room temperature for 10min and then centrifuged at 1500 rpm for 10 min at 4°C. The supernatant was collected and diluted 2-fold using standard PBS pH ~7.5 before flash-frozen in liquid nitrogen and stored at -80°C. Serum levels (in duplicates) of the cytokines were determined using Luminex 200 platform and mouse cytokine/chemokine 32-plex discovery assay (MD32) by Eve Technologies (Calgary, AB, Canada). Raw values for each group were normalized to sham stroke controls.

SUPPLEMENTARY REFERENCES

1 Zack GW, Rogers WE, Latt SA. Automatic measurement of sister chromatid exchange frequency. *J Histochem Cytochem* 1977; **25**: 741–753.

2 Shanina EV, Schallert T, Witte OW, Redecker C. Behavioral recovery from unilateral photothrombotic infarcts of the forelimb sensorimotor cortex in rats: Role of the contralateral cortex. *Neuroscience* 2006; **139**: 1495–1506.

3 Sweetnam D, Holmes A, Tennant KA, Zamani A, Walle M, Jones P *et al.* Diabetes Impairs Cortical Plasticity and Functional Recovery Following Ischemic Stroke. *J Neurosci* 2012; **32**: 5132–5143.

4 Tennant KA, Jones TA. Sensorimotor Behavioral Effects of Endothelin-1 Induced Small Cortical Infarcts in C57BL/6 Mice. *J Neurosci Methods* 2009; **181**: 18–26.

5 Morris R. Developments of a water-maze procedure for studying spatial learning in the rat. *J Neurosci Methods* 1984; **11**: 47–60.

6 Vorhees CV, Williams MT. Morris water maze: procedures for assessing spatial and related forms of learning and memory. *Nat Protoc* 2006; **1**: 848–858.

7 Seibenhener ML, Wooten MC. Use of the Open Field Maze to Measure Locomotor and Anxiety-like Behavior in Mice. *J Vis Exp JoVE* 2015; : 52434.

8 Brown CE, Aminoltejari K, Erb H, Winship IR, Murphy TH. In Vivo Voltage-Sensitive Dye Imaging in Adult Mice Reveals That Somatosensory Maps Lost to Stroke Are Replaced over Weeks by New Structural and Functional Circuits with Prolonged Modes of Activation within Both the Peri-Infarct Zone and Distant Sites. *J Neurosci* 2009; **29**: 1719–1734.

9 Motaharinia M, Gerrow K, Boghozian R, White E, Choi S-E, Delaney KR *et al.* Longitudinal functional imaging of VIP interneurons reveals sup-population specific effects of stroke that are rescued with chemogenetic therapy. *Nat Commun* 2021; **12**: 6112.

10 Reeson P, Tennant KA, Gerrow K, Wang J, Novak SW, Thompson K *et al.* Delayed Inhibition of VEGF Signaling after Stroke Attenuates Blood–Brain Barrier Breakdown and Improves Functional Recovery in a Comorbidity-Dependent Manner. *J Neurosci* 2015; **35**: 5128–5143.

Supp Figure 1

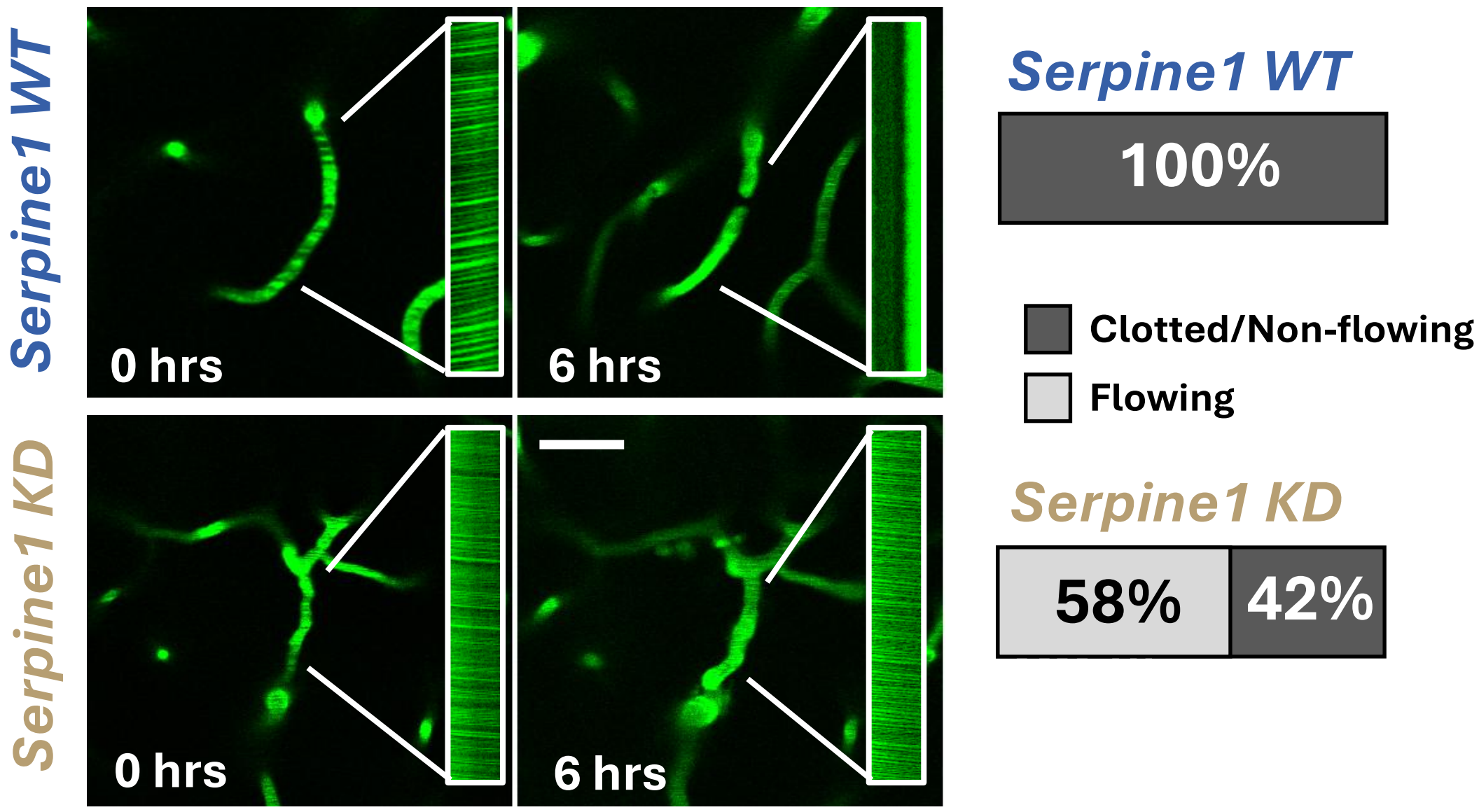

Supp Fig. 1. *In vivo* two-photon images with accompanying line scans of a capillary pre- and post-bleed (ie. 6hrs post-bleed) in *Serpine1* WT and KD groups (n=10 and 24 capillaries, respectively). In *Serpine1* WT group, all (100%) of the vessels were stalled at 6 hours, whereas in *Serpine1* KD group only 42% were stalled and 58% were flowing. Scale bar = 25µm.

Supp Figure 2

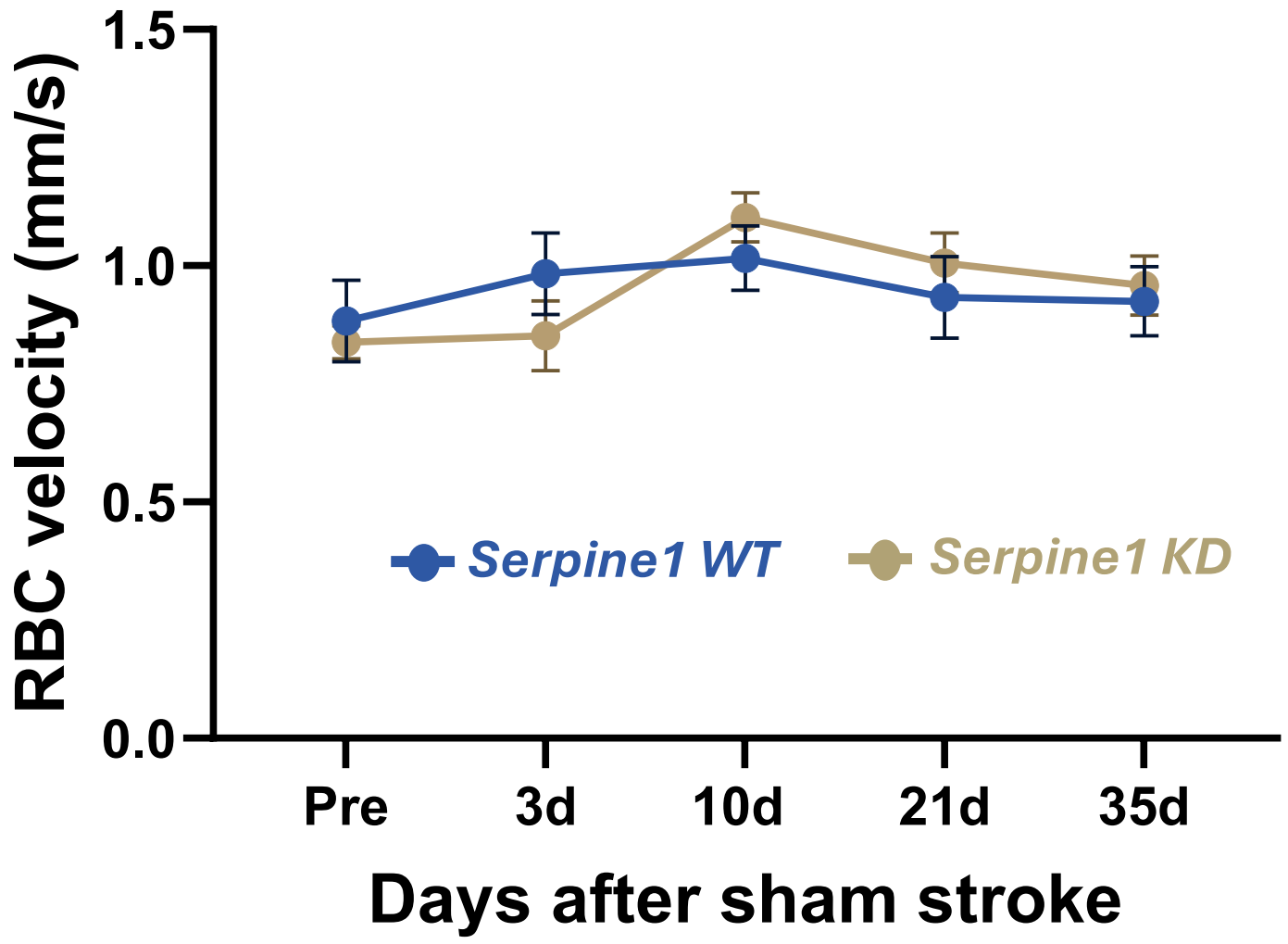

Supp Fig. 2. Data show RBC velocities over time in *Serpine1* WT and KD mice (n=3 from each genotype) after sham stroke. RBC velocities did not differ between these groups (2-way ANOVA, Main effect of Genotype: $F_{(1, 560)}$ = 0.004, =*0.94*; Main effect of Time: $F_{(4, 560)}$ = 2.11 *p* = 0.07; Genotype x Time Interaction: $F_{(4, 560)}$ = 0.84, *p*=0.50). Data expressed as the mean ± SD.

Supp Figure 3

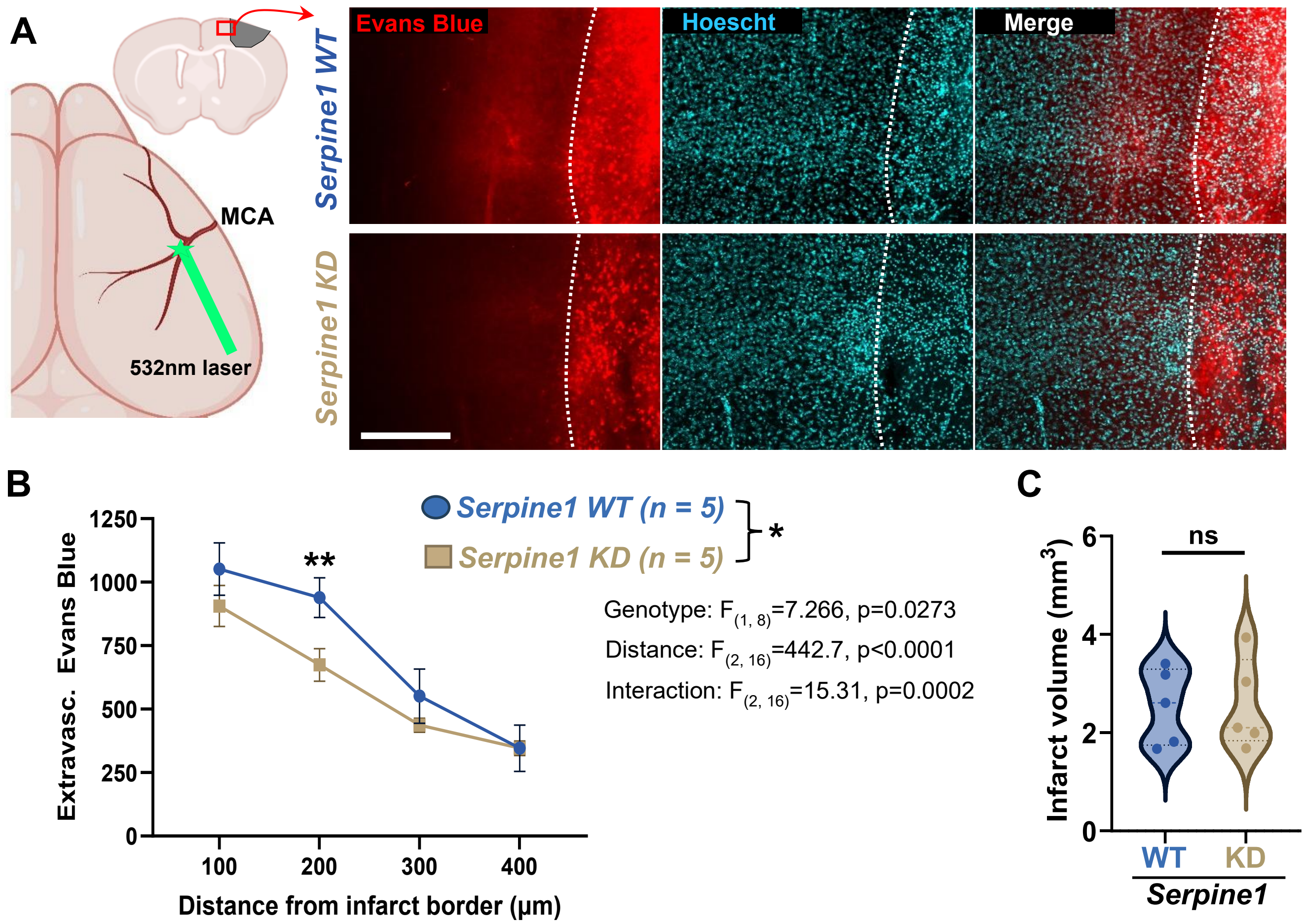

Supp Fig. 3. A. Left: Cartoon showing photothrombotic stroke targeting a distal branch of the MCA. **Right:** Confocal images showing extravasation of Evan's Blue dye in peri-infarct cortex 3 days after stroke in *Serpine1* WT and KD mice. Hoescht (Cyan) staining helped determine the infarct border (white dashed line). Scale bar = 200µm. **B.** Graph shows genotype differences in extravasation of Evans Blue dye (normalized to contralateral hemisphere) as a function of distance from infarct border (µm). Statistics related to main effects and interaction from a 2-way ANOVA are shown. **C.** Infarct volume did not significantly differ between genotypes (p=0.1738). Data expressed as the mean ± SD.

Supp Figure 4

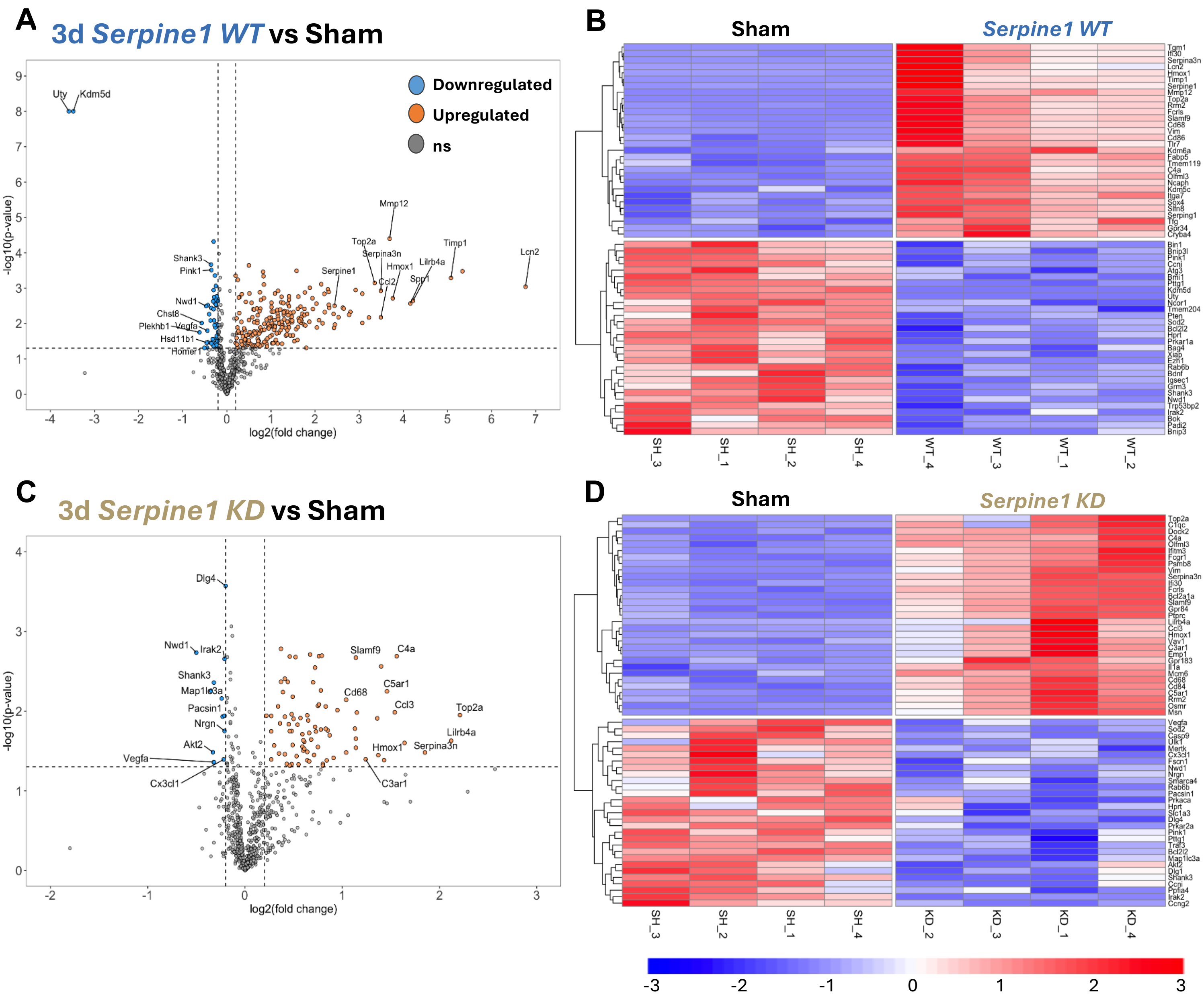

Supp Fig. 4. Volcano plot depicting differentially expressed genes (DEG) in **A.** *Serpine1* WT vs. sham, and **C.** *Serpine1* KD vs. sham as a function of $\log_2$ fold change (fc) and statistical significance of $p < 0.05$, denoted in y-axis as $-\log_{10}$(p-value). Downregulated DEGs shown in blue, upregulated in orange, and non-significant in grey. Top 10 up- and down-regulated DEG are labelled. Heatmap (corresponding to volcano plot) of the top 30 regulated DEG in B. *Serpine1* WT vs. sham, and D. *Serpine1* KD vs sham. Red columns indicate upregulated (*3* on scaled expression), and blue columns indicate downregulated (*-3* on scaled expression). n = 4 per group (sham, *Serpine1* WT and KD).

Supp Figure 5

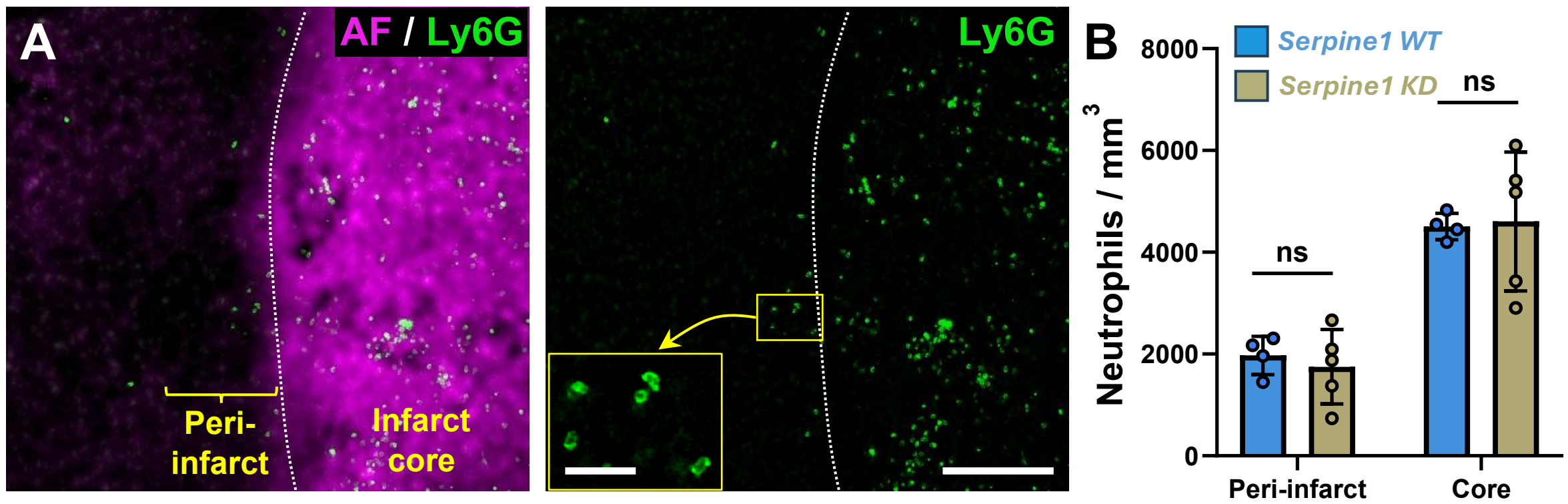

Supp Fig. 5. A. Confocal images of a coronal section taken 3 days after stroke showing Ly6G labelled neutrophils (green) overlaid with autofluorescence (AF, magenta) images delineating the infarct boundary. *Serpine1* WT = 4 mice, *Serpine1* KD = 5 mice. **B.** Graphs show density of Ly6G labelled neutrophils in Peri-infarct and infarct core regions. Data analyzed with two-tailed unpaired t-test. ns = non-significant. Data expressed as mean ± SD. Scale bar in A = 200μm, inset = 50μm.

Supp Figure 6

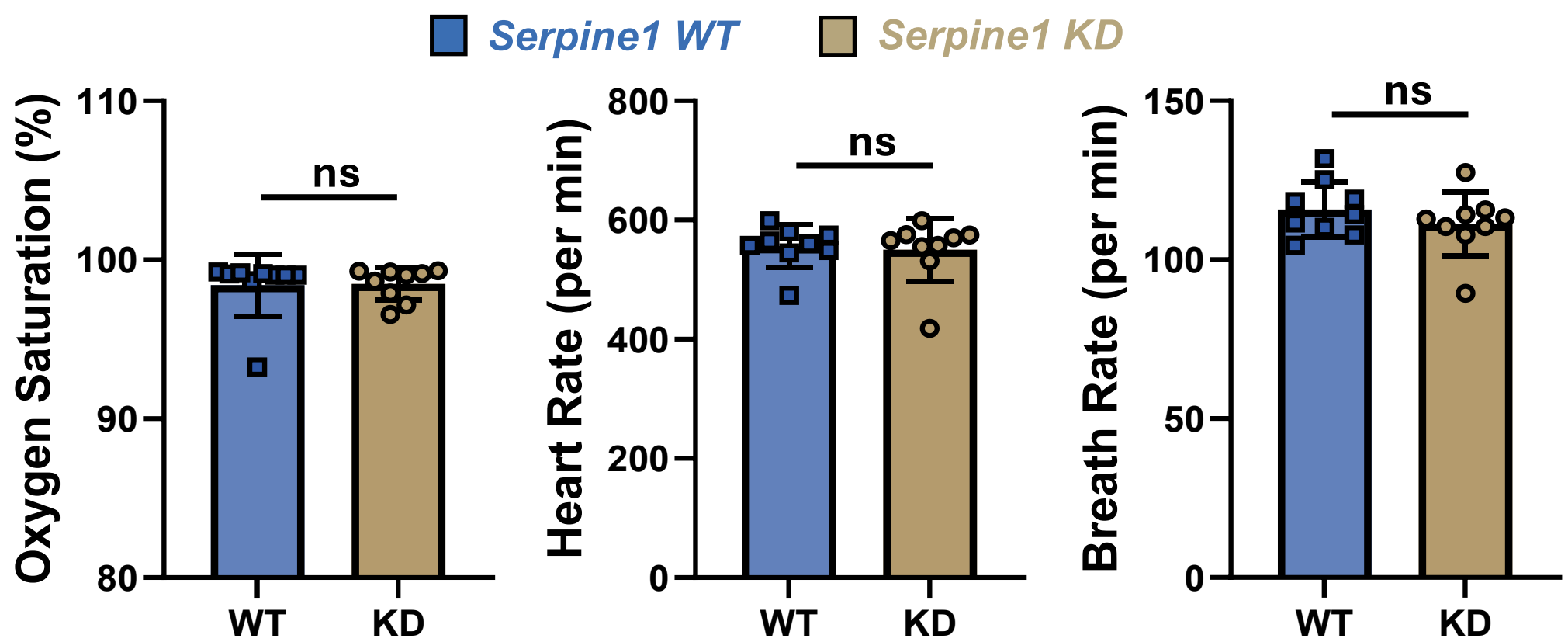

Supp Fig. 6. Analysis of mouse vital signs including oxygen saturation (%), heart rate (beats per min), and breath rate (per min) in *Serpine1* WT and KD mice 3d post stroke. Data analyzed with 2-tailed unpaired t-test. ns = non-significant. Data expressed as the mean ± SD.

Supp Figure 7

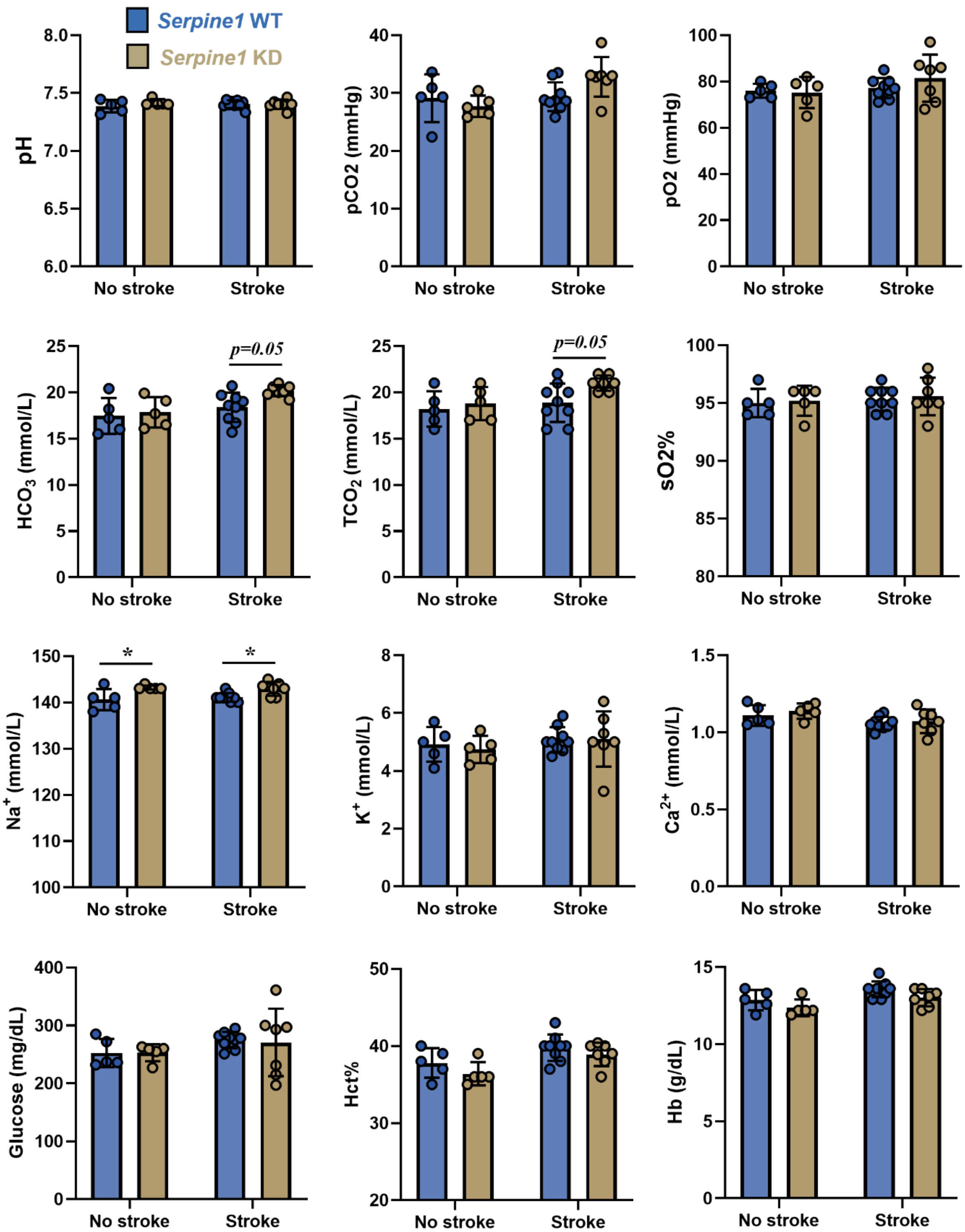

Supp Fig. 7. Analysis of blood chemistry in *Serpine1* WT and KD mice in sham controls and 3d post stroke. Data analyzed with two-way ANOVA and Sidak's multiple comparisons test. *$p<0.05$. Data expressed as the mean ± SD.

Supp Figure 8

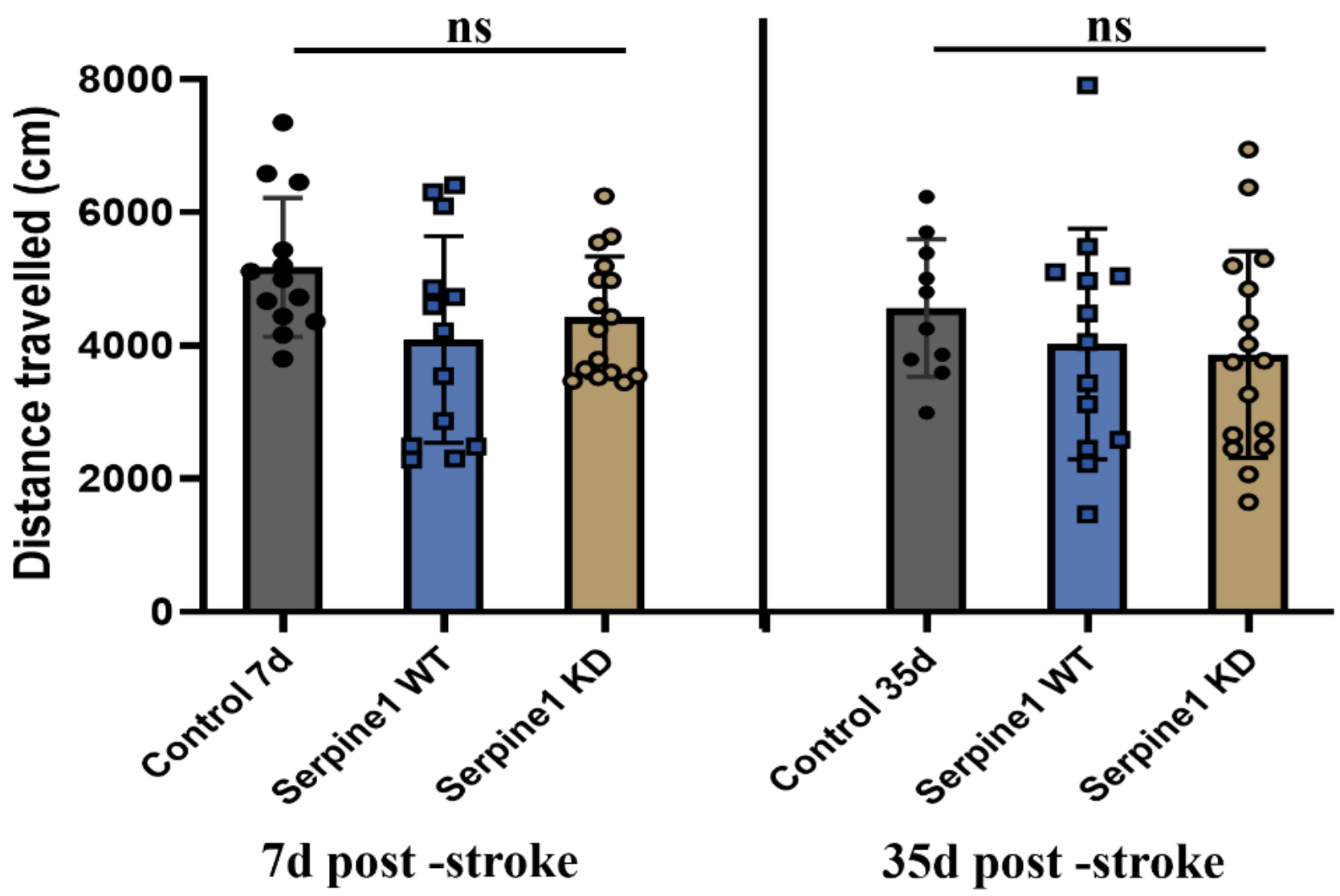

Supp Fig. 8. Open field behavior test between sham, *Serpine1* WT and KD mice performed at 7d and 35d post-stroke. Data analyzed with two-way ANOVA and Tukey's multiple comparisons test. ns = non-significant. Data expressed as the mean ± SD.

SUPPLEMENTARY TABLE 1

Figures	Experiment	# mice excluded	Reason
**1**	Serpine1/PAI-1 immunostaining	2	Inadequate stroke induction
**1**	Nanostring	0	
**1**	Infarct volume	2	Inadequate stroke induction
		1	Humane endpoint reached
**2 & 3**	2P imaging	3	Inadequate stroke induction
		5	Stroke disrupted optical clarity of window
		10	Cranial window detached
		2	Humane endpoint reached
**3**	rPAI-1	1	Cranial window detached
**4**	BBB	1	Humane endpoint reached
**5**	Nanostring	0	
**6**	IOS	5	Stroke disrupted optical clarity of window
**7**	Behaviour	2	Humane endpoint reached
**Total**		**34**	
